# Supplementary material for: The Association between Gestational Diabetes and the Microbiome: A Systematic Review and Meta-Analysis
Source: Microorganisms. 2023 Jul 4;11(7):1749. doi: 10.3390/microorganisms11071749 (PMC10385443; doi:10.3390/microorganisms11071749)
Supplement: Supplementary file 1 [file microorganisms-11-01749-s001.zip › microorganisms-2483000-supplementary.pdf]

Supplementary material

Supplementary Material File S1 - PRISMA 2020 Checklist

Supplementary Material File S2 - Meta-analysis results: Funnel and Forest Plots

Supplementary Material File S3 - Article Bias Evaluation - National Institute of Health

Reporting guidelines: Supplementary Material File S1 - PRISMA 2020 Checklist

PRISMA 2020 Checklist

Section and Topic	Item #	Checklist item	Location where item is reported
**TITLE**			
Title	1	Identify the report as a systematic review.	Page 1 "The association between gestational diabetes and the microbiome: a systematic review and meta-analysis"
**ABSTRACT**			
Abstract	2	See the PRISMA 2020 for Abstracts checklist.	Supplementary Material File S2
**INTRODUCTION**			
Rationale	3	Describe the rationale for the review in the context of existing knowledge.	Page 1 "While the exact mechanisms underlying the development of GDM are not entirely understood, recent evidence suggests that the microbiome may play a relevant role."
Objectives	4	Provide an explicit statement of the objective(s) or question(s) the review addresses.	Page 2: "This systematic review with meta-analysis aims to update the previous reviews on this topic [21,22], including the new data available,

			summarizing, and quantifying all relevant information to describe the current state of knowledge regarding the relationship between the microbiome and GDM. This review provides a comprehensive summary of the evidence on this topic, identifies areas of agreement and disagreement in the literature, and suggests directions for future research. The authors conducted a comprehensive literature search to identify relevant studies and used meta-analytic techniques to synthesise these studies' findings and assess the evidence's overall strength to achieve these goals. The objective of this systematic review with meta-analysis was to summarize the existing evidence on the differences in microbiota composition in pregnant women with gestational diabetes compared to healthy pregnancies.”
**METHODS**			
Eligibility criteria	5	Specify the inclusion and exclusion criteria for the review and how studies were grouped for the syntheses.	Page 2: “As inclusion criteria were defined, studies presented data on the relative abundance of the microbiome in both gestational diabetes and control groups. Conversely, it was defined as exclusion criteria 1) if the studies were reviews or systematic reviews; 2) comprised subjects that were not humans, such as animals or cells; 3) if the study did not have a control group without gestational diabetes; 4) contained incomplete data, such as standard deviation or interval range or 5) did not use comprehensive methods. ”

Information sources	6	Specify all databases, registers, websites, organisations, reference lists and other sources searched or consulted to identify studies. Specify the date when each source was last searched or consulted.	Pages 2: “A comprehensive search of the literature was conducted on November 13th, 2022, aiming to identify relevant studies on the relationship between the microbiome and gestational diabetes mellitus. The search was conducted in PubMed, Scopus, and Web of Science databases. The search was not limited to studies published in a specific language or time period. The QUERY utilized was (Microbiome [MeSH] AND gestational diabetes [MeSH]) OR (Microbiota [MeSH] AND gestational diabetes [MeSH]). The reference lists of identified studies and relevant review articles were also analyzed for additional studies.”
Search strategy	7	Present the full search strategies for all databases, registers and websites, including any filters and limits used.	Pages 2: “A comprehensive search of the literature was conducted on November 13th, 2022, aiming to identify relevant studies on the relationship between the microbiome and gestational diabetes mellitus. The search was conducted in PubMed, Scopus, and Web of Science databases. The search was not limited to studies published in a specific language or time period. The QUERY utilized was (Microbiome [MeSH] AND gestational diabetes [MeSH]) OR (Microbiota [MeSH] AND gestational diabetes [MeSH]). The reference lists of identified studies and relevant review articles were also analyzed for additional studies.”
Selection process	8	Specify the methods used to decide whether a study met the inclusion criteria of the review, including how many reviewers screened each record and each report retrieved, whether they worked independently, and if applicable, details of automation tools used in the process.	Page 3: “All the articles were screened by four researchers independently and blindly. The studies were selected based on titles and abstracts in the first phase. The full text was analyzed in a second moment, selecting the relevant articles. Studies that led to inconsistencies in the decision were reanalyzed by all researchers, who provided their reasons for inclusion

			or exclusion until a consensus was reached. Studies referring to maternal gut microbiota and mycobiota, fetal gut microbiota, meconium's microbiota, placental microbiota, vaginal microbiota, maternal oral microbiota, fetal oral microbiota, and colostrum microbiota were included. "
Data collection process	9	Specify the methods used to collect data from reports, including how many reviewers collected data from each report, whether they worked independently, any processes for obtaining or confirming data from study investigators, and if applicable, details of automation tools used in the process.	Page 3: "Using a self-developed data extraction table to extract data, the articles were screened to retrieve the following information on the study characteristics: 1) Title of the article, authors, publication year, and journal; 2) Site of microbiome collection; 3) Sample size; 4) Study population; 5) Method used to identify microbiota; 6) Gene sequencing region analyzed; 7) Moment when the sample was collected; and 8) Taxonomic level of the results presented (e.g., phylum, genus).<br>In a second phase, the same process was used to organize the information on the mean or median of relative abundances of each microbe in the gestational diabetes group and control and respective standard deviation or interval range. The information on this phase was obtained either directly from the article or its supplementary material by contacting the corresponding author. When the information was only available in an image, the underlying numerical data was extracted using WebPlotDigitizer: Version 4.6 software. Three researchers measured the figures independently, and the results were compared to guarantee accuracy."

Data items	10a	List and define all outcomes for which data were sought. Specify whether all results that were compatible with each outcome domain in each study were sought (e.g., for all measures, time points, analyses), and if not, the methods used to decide which results to collect.	Page 3: "Using a self-developed data extraction table to extract data, the articles were screened to retrieve the following information on the study characteristics: 1) Title of the article, authors, publication year, and journal; 2) Site of microbiome collection; 3) Sample size; 4) Study population; 5) Method used to identify microbiota; 6) Gene sequencing region analyzed; 7) Moment when the sample was collected; and 8) Taxonomic level of the results presented (e.g., phylum, genus).<br>In a second phase, the same process was used to organize the information on the mean or median of relative abundances of each microbe in the gestational diabetes group and control and respective standard deviation or interval range. The information on this phase was obtained either directly from the article or its supplementary material by contacting the corresponding author. When the information was only available in an image, the underlying numerical data was extracted using WebPlotDigitizer: Version 4.6 software. Three researchers measured the figures independently, and the results were compared to guarantee accuracy."
	10b	List and define all other variables for which data were sought (e.g., participant and intervention characteristics, funding sources). Describe any assumptions made about any missing or unclear information.	Page 3: "Using a self-developed data extraction table to extract data, the articles were screened to retrieve the following information on the study characteristics: 1) Title of the article, authors, publication year, and journal; 2) Site of microbiome collection; 3) Sample size; 4) Study population; 5) Method used to identify microbiota; 6) Gene sequencing region analyzed; 7) Moment when the sample was collected; and 8)

			Taxonomic level of the results presented (e.g., phylum, genus).<br>In a second phase, the same process was used to organize the information on the mean or median of relative abundances of each microbe in the gestational diabetes group and control and respective standard deviation or interval range. The information on this phase was obtained either directly from the article or its supplementary material by contacting the corresponding author. When the information was only available in an image, the underlying numerical data was extracted using WebPlotDigitizer: Version 4.6 software. Three researchers measured the figures independently, and the results were compared to guarantee accuracy.”
Study risk of bias assessment	11	Specify the methods used to assess risk of bias in the included studies, including details of the tool(s) used, how many reviewers assessed each study and whether they worked independently, and if applicable, details of automation tools used in the process.	Page 4: “The individual quality and risk of bias of each study included were evaluated using study quality assessment tools of the National Heart, Lung, and Blood Institute of the National Institutes of Health (NIH) for quality assessment of Observational Cohort and Cross-Sectional Studies and of Case-Control Studies. The studies’ quality was accessed by three investigators independently. The results were compared, and inconsistencies were debated until a consensus was reached. This information can be found in Supplementary Material 3.”
Effect measures	12	Specify for each outcome the effect measure(s) (e.g., risk ratio, mean difference) used in the synthesis or presentation of results.	Page 3: “In a second phase, the same process was used to organize the information on the mean or median of relative abundances of each microbe in the gestational diabetes group and control and respective standard deviation or interval range.”

Synthesis methods	13a	Describe the processes used to decide which studies were eligible for each synthesis (e.g., tabulating the study intervention characteristics and comparing against the planned groups for each synthesis (item #5)).	Page 3: "Using a self-developed data extraction table to extract data, the articles were screened to retrieve the following information on the study characteristics: 1) Title of the article, authors, publication year, and journal; 2) Site of microbiome collection; 3) Sample size; 4) Study population; 5) Method used to identify microbiota; 6) Gene sequencing region analyzed; 7) Moment when the sample was collected; and 8) Taxonomic level of the results presented (e.g., phylum, genus)."
	13b	Describe any methods required to prepare the data for presentation or synthesis, such as handling of missing summary statistics, or data conversions.	Page 3: "In a second phase, the same process was used to organize the information on the mean or median of relative abundances of each microbe in the gestational diabetes group and control and respective standard deviation or interval range."
	13c	Describe any methods used to tabulate or visually display results of individual studies and syntheses.	Page 3: "In a second phase, the same process was used to organize the information on the mean or median of relative abundances of each microbe in the gestational diabetes group and control and respective standard deviation or interval range."
	13d	Describe any methods used to synthesize results and provide a rationale for the choice(s). If meta-analysis was performed, describe the model(s), method(s) to identify the presence and extent of statistical heterogeneity, and software package(s) used.	Page 3: "RevMan 5.4.1 [25] was used to analyze the data extracted by elaborating meta-analysis and their respective forest plots. Meta-analysis was performed using the inverse variance with a random-effects model to calculate the standardized mean differences with 95% confidence intervals (CI). A random-effects model was used to estimate the pooled effect size due to the expected heterogeneity among studies. The heterogeneity among studies was evaluated by Tau^2, and I^2 Statistics. A P-value < 0.05 was considered statistically significant. A Z-test to test for overall effect

			size. A P-value < 0.05 was considered statistically significant.<br>Body sites without enough quantitative data to enable meta-analysis were not further considered. The meta-analysis was limited to articles that reported values for standard deviation or other statistical measures that could be converted to standardized effect sizes. Articles that did not meet this criterion were excluded from the analysis to ensure the accuracy and validity of the meta-analytic findings. Forest plots were extracted after analysis, as well as funnel plots. ”
	13e	Describe any methods used to explore possible causes of heterogeneity among study results (e.g., subgroup analysis, meta-regression).	Not applicable
	13f	Describe any sensitivity analyses conducted to assess robustness of the synthesized results.	Page 3: “RevMan 5.4.1 [25] was used to analyze the data extracted by elaborating meta-analysis and their respective forest plots. Meta-analysis was performed using the inverse variance with a random-effects model to calculate the standardized mean differences with 95% confidence intervals (CI). A random-effects model was used to estimate the pooled effect size due to the expected heterogeneity among studies. The heterogeneity among studies was evaluated by Tau^2, and I^2 Statistics. A P-value < 0.05 was considered statistically significant. A Z-test to test for overall effect size. A P-value < 0.05 was considered statistically significant.”
Reporting bias assessment	14	Describe any methods used to assess risk of bias due to missing results in a synthesis (arising from reporting biases).	Page 3: “Forest plots were extracted after analysis, as well as funnel plots.”

Certainty assessment	15	Describe any methods used to assess certainty (or confidence) in the body of evidence for an outcome.	Page 3: "RevMan 5.4.1 [25] was used to analyze the data extracted by elaborating meta-analysis and their respective forest plots. Meta-analysis was performed using the inverse variance with a random-effects model to calculate the standardized mean differences with 95% confidence intervals (CI). A random-effects model was used to estimate the pooled effect size due to the expected heterogeneity among studies. The heterogeneity among studies was evaluated by Tau^2, and I^2 Statistics. A P-value < 0.05 was considered statistically significant. A Z-test to test for overall effect size. A P-value < 0.05 was considered statistically significant."
**RESULTS**			
Study selection	16a	Describe the results of the search and selection process, from the number of records identified in the search to the number of studies included in the review, ideally using a flow diagram.	Figure 1
	16b	Cite studies that might appear to meet the inclusion criteria, but which were excluded, and explain why they were excluded.	Page 4: "Of the remaining articles, 21 were included in the meta-analysis. The reasons for exclusion of the remaining 156 articles were: 29 did not present relevant data, 19 did not have enough similar studies to enable the meta-analysis, 24 were not original articles, 6 studied an incorrect population (1 studied only obese women, 4 did not have a control group, 1 studied only 5 years postpartum), 70 did not address the research question, 3 did not study humans and 5 used only PCR techniques to measure relative abundance."

Study characteristics	17	Cite each included study and present its characteristics.	Table 1
Risk of bias in studies	18	Present assessments of risk of bias for each included study.	Supplementary Material 3 - Article Bias
Results of individual studies	19	For all outcomes, present, for each study: (a) summary statistics for each group (where appropriate) and (b) an effect estimate and its precision (e.g., confidence/credible interval), ideally using structured tables or plots.	Supplementary Material 2 - Meta-analysis
Results of syntheses	20a	For each synthesis, briefly summarize the characteristics and risk of bias among contributing studies.	Supplementary Material File S2 - Meta-analysis and Supplementary Material File S3 - Article Bias Evaluation - National Institute of Health
	20b	Present results of all statistical syntheses conducted. If meta-analysis was done, present for each the summary estimate and its precision (e.g., confidence/credible interval) and measures of statistical heterogeneity. If comparing groups, describe the direction of the effect.	Supplementary Material File S2 - Meta-analysis
	20c	Present results of all investigations of possible causes of heterogeneity among study results.	NA
	20d	Present results of all sensitivity analyses conducted to assess the robustness of the synthesized results.	Supplementary Material File S2 - Meta-analysis
Reporting biases	21	Present assessments of risk of bias due to missing results (arising from reporting biases) for each synthesis assessed.	Supplementary Material File S2 - Meta-analysis

Certainty of evidence	22	Present assessments of certainty (or confidence) in the body of evidence for each outcome assessed.	Supplementary Material File S2 - Meta-analysis
**DISCUSSION**			
Discussion	23a	Provide a general interpretation of the results in the context of other evidence.	Pages 9 - 11. For example: "Classically, Bacteroidetes and Firmicutes represent 90% of the gut's microbiota, which could mostly be confirmed by the relative abundance of data collected throughout the analyzed studies. Moreover, the Firmicutes/Bacteroidetes ratio is often used as a marker of dysbiosis. Despite this, it was not possible to find statistically significant differences in both phyla between the GDM and the control group."
	23b	Discuss any limitations of the evidence included in the review.	Pages 11-12: For example, "It is important to highlight that these findings are based on observational studies. Therefore, it is not possible to establish a causal or consequential relationship between microbiota and metabolic outcomes in women with GDM. (…)<br>Finally, the lack of quantitative data also led to occasional assumptions being made from figures presented instead of values provided by authors. Although this process is considered accurate, perhaps some values can differ from the original ones."
	23c	Discuss any limitations of the review processes used.	Page 11-12: "It is worth noting that this decision has its limitations. Even with broader methods, biases may still be introduced by differences in sampling or analysis techniques across studies (65). However, this approach was the most appropriate for the goals of our review and provides a solid foundation for further research in this area.

			Finally, the lack of quantitative data also led to occasional assumptions being made from figures presented instead of values provided by authors. Although this process is considered accurate, perhaps some values can differ from the original ones.”
	23d	Discuss implications of the results for practice, policy, and future research.	Page 12: “Furthermore, considerable heterogeneity was observed throughout the meta-analysis, underscoring the potential benefits of adhering to international protocols to standardize the findings and facilitate their interpretation and comparison. Overall, while the present review provides valuable insights into the association between GDM and microbiota of different body sites, future research should focus on generating and sharing more robust quantitative data with standardizing methodologies to enhance the comparability of results across studies. An association would be established, allowing advantages to be taken from a clinical actuation perspective to minimize effects on the cause or consequences of this relation.”
**OTHER INFORMATION**			
Registration and protocol	24a	Provide registration information for the review, including register name and registration number, or state that the review was not registered.	NA
	24b	Indicate where the review protocol can be accessed, or state that a protocol was not prepared.	NA

	24c	Describe and explain any amendments to information provided at registration or in the protocol.	NA
Support	25	Describe sources of financial or non-financial support for the review, and the role of the funders or sponsors in the review.	Page 12: "This research was funded by national funds through FCT - Portuguese Foundation for Science and Technology, under the scope of the Cardiovascular R&D Center – UnIC (UIDB/00051/2020 and UIDP/00051/2020)."
Competing interests	26	Declare any competing interests of review authors.	Page 12: "The authors declare no conflict of interests."
Availability of data, code and other materials	27	Report which of the following are publicly available and where they can be found: template data collection forms; data extracted from included studies; data used for all analyses; analytic code; any other materials used in the review.	Page 12: "The data presented in this study are available in the supplementary material."

From: Page MJ, McKenzie JE, Bossuyt PM, Boutron I, Hoffmann TC, Mulrow CD, et al. The PRISMA 2020 statement: an updated guideline for reporting systematic reviews. BMJ 2021;372:n71. doi: 10.1136/bmj.n71

For more information, visit: http://www.prisma-statement.org/

PRISMA 2020 for Abstracts

Section and Topic	Item #	Checklist item	Reported (Yes/No)
**TITLE**			
Title	1	Identify the report as a systematic review.	Yes

**BACKGROUND**			
Objectives	2	Provide an explicit statement of the main objective(s) or question(s) the review addresses.	Yes
**METHODS**			
Eligibility criteria	3	Specify the inclusion and exclusion criteria for the review.	No*
Information sources	4	Specify the information sources (e.g., databases, registers) used to identify studies and the date when each was last searched.	Yes
Risk of bias	5	Specify the methods used to assess risk of bias in the included studies.	No*
Synthesis of results	6	Specify the methods used to present and synthesise results.	Yes
**RESULTS**			
Included studies	7	Give the total number of included studies and participants and summarise relevant characteristics of studies.	Yes
Synthesis of results	8	Present results for main outcomes, preferably indicating the number of included studies and participants for each. If meta-analysis was done, report the summary estimate and confidence/credible interval. If comparing groups, indicate the direction of the effect (i.e., which group is favoured).	Yes
**DISCUSSION**			
Limitations of evidence	9	Provide a brief summary of the limitations of the evidence included in the review (e.g., study risk of bias, inconsistency and imprecision).	Yes
Interpretation	10	Provide a general interpretation of the results and important implications.	Yes

**OTHER**			
Funding	11	Specify the primary source of funding for the review.	Yes
Registration	12	Provide the register name and registration number.	NA

From: Page MJ, McKenzie JE, Bossuyt PM, Boutron I, Hoffmann TC, Mulrow CD, et al. The PRISMA 2020 statement: an updated guideline for reporting systematic reviews. BMJ 2021;372:n71. doi: 10.1136/bmj.n71

* The omission of these details in the abstract is attributed to the inherent constraint of limited word count. However, comprehensive access to these specificities can be found within the "Materials and Methods" section, readily available for consultation.

For more information, visit: http://www.prisma-statement.org/

Supplementary File S2 Meta-analysis: Forest and Funnel Plots

Placenta 16

Phylum 16

Genus 18

Gut 20

Phylum 20

Genus 25

Meconium 33

Phylum 33

Genus 37

Placenta

Phylum

Actinobacteria

Study or Subgroup	GDM Mean	GDM SD	GDM Total	Control Mean	Control SD	Control Total	Weight	Std. Mean Difference IV, Random, 95% CI	Year
Bassols 2016	2.5324	3.9801	50	2.8061	3.3902	157	81.6%	-0.08 [-0.40, 0.24]	2016
Olomu 2020	8.778	21.436	10	2.372	3.323	10	10.5%	0.40 [-0.49, 1.29]	2020
Tang 2020	9.4	2.146	8	10	1.897	7	7.9%	-0.28 [-1.30, 0.74]	2020
**Total (95% CI)**			**68**			**174**	**100.0%**	**-0.04 [-0.33, 0.24]**	

Heterogeneity: Tau² = 0.00; Chi² = 1.20, df = 2 (P = 0.55); I² = 0%
Test for overall effect: Z = 0.29 (P = 0.77)

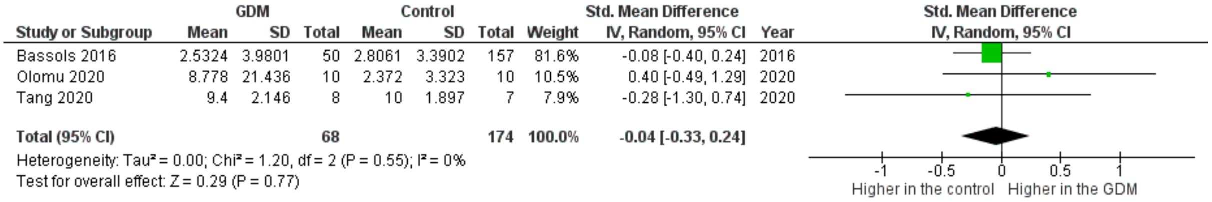

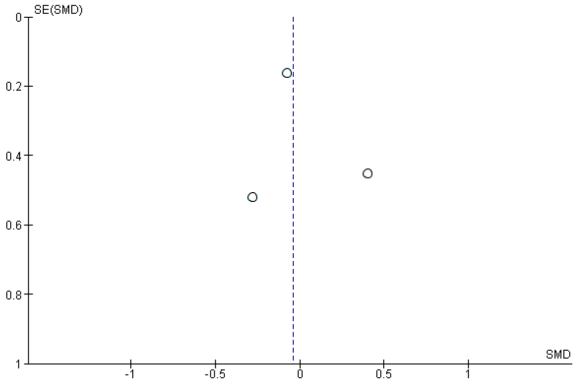

Bacteroidetes

Study or Subgroup	GDM Mean	GDM SD	GDM Total	Control Mean	Control SD	Control Total	Weight	Std. Mean Difference IV, Random, 95% CI	Year
Bassols 2016	15.0052	5.8543	50	17.4253	8.556	157	81.2%	-0.30 [-0.62, 0.02]	2016
Olomu 2020	10.4768	24.316	10	7.079	8.5516	10	10.7%	0.18 [-0.70, 1.06]	2020
Tang 2020	42.1	24.007	8	39.4	25.735	7	8.0%	0.10 [-0.91, 1.12]	2020
**Total (95% CI)**			**68**			**174**	**100.0%**	**-0.22 [-0.51, 0.07]**	

Heterogeneity: Tau² = 0.00; Chi² = 1.43, df = 2 (P = 0.49); I² = 0%
Test for overall effect: Z = 1.48 (P = 0.14)

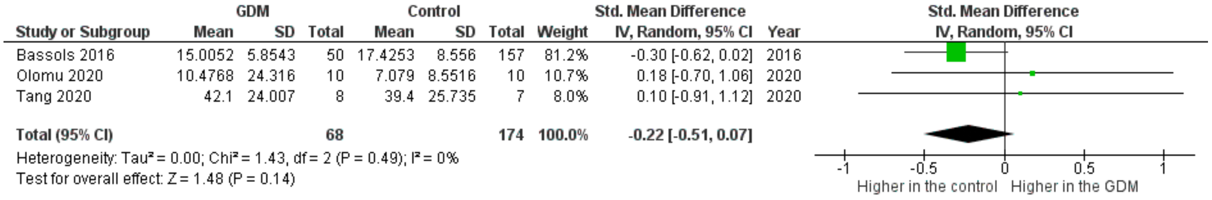

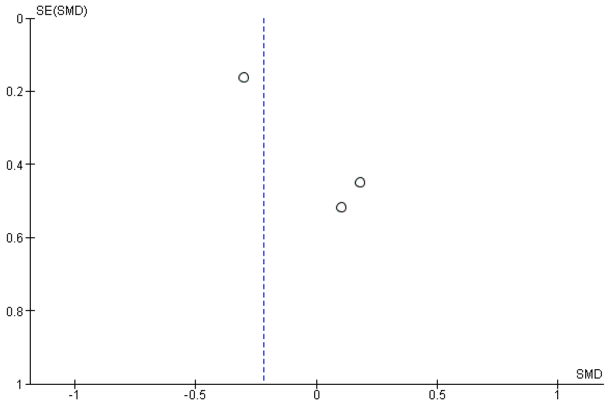

Firmicutes

Study or Subgroup	GDM Mean	GDM SD	GDM Total	Control Mean	Control SD	Control Total	Weight	Std. Mean Difference IV, Random, 95% CI	Year
Bassols 2016	15.7812	14.1819	50	8.7128	8.0608	157	73.1%	0.71 [0.39, 1.04]	2016
Olomu 2020	10.4768	24.316	10	7.079	8.5516	10	15.3%	0.18 [-0.70, 1.06]	2020
Tang 2020	42.1	24.007	8	39.4	25.735	7	11.7%	0.10 [-0.91, 1.12]	2020
**Total (95% CI)**			**68**			**174**	**100.0%**	**0.56 [0.20, 0.92]**	

Heterogeneity: Tau² = 0.02; Chi² = 2.27, df = 2 (P = 0.32); I² = 12%
Test for overall effect: Z = 3.06 (P = 0.002)

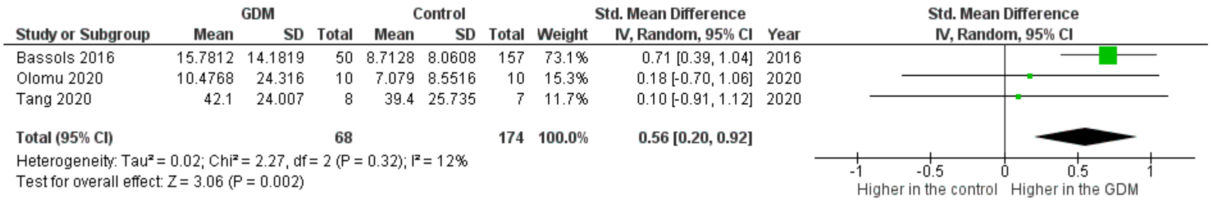

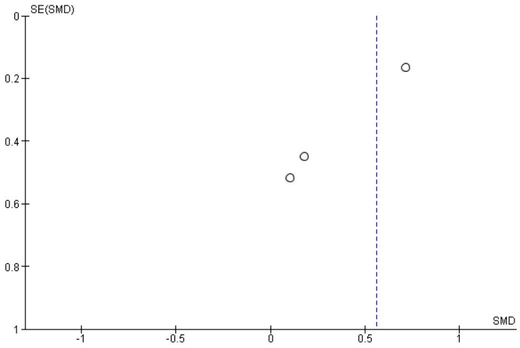

Proteobacteria

Study or Subgroup	GDM Mean	GDM SD	GDM Total	Control Mean	Control SD	Control Total	Weight	Std. Mean Difference IV, Random, 95% CI	Year
Bassols 2016	64.1911	17.5648	50	69.6479	12.0484	157	81.2%	-0.40 [-0.72, -0.08]	2016
Olomu 2020	1.63069	5.0755	10	0.99418	1.1339	10	10.8%	0.17 [-0.71, 1.04]	2020
Tang 2020	5.6	1.893	8	7.1	5.596	7	8.0%	-0.35 [-1.37, 0.68]	2020
**Total (95% CI)**			**68**			**174**	**100.0%**	**-0.34 [-0.62, -0.05]**	

Heterogeneity: Tau² = 0.00; Chi² = 1.41, df = 2 (P = 0.49); I² = 0%
Test for overall effect: Z = 2.27 (P = 0.02)

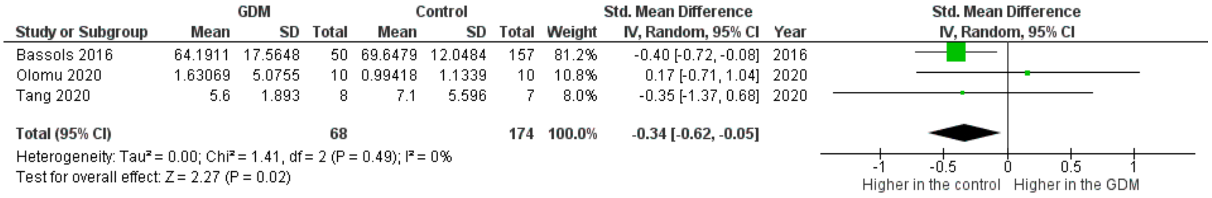

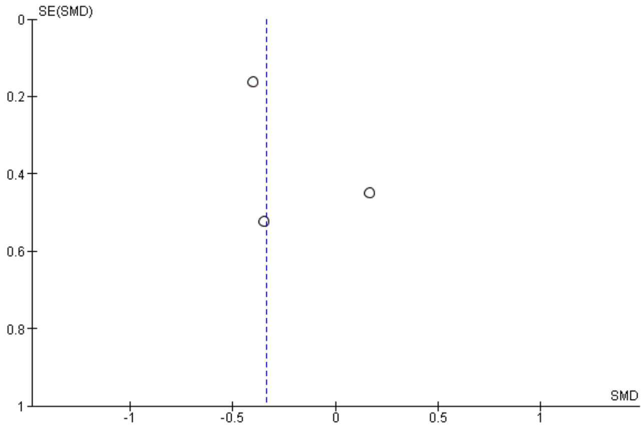

Genus

Faecalibacterium

Study or Subgroup	GDM Mean	GDM SD	GDM Total	Control Mean	Control SD	Control Total	Weight	Std. Mean Difference IV, Random, 95% CI	Year
Bassols 2016	0.967	3.2073	50	0.0054	0.016	157	69.7%	0.61 [0.29, 0.93]	2016
Olomu 2020	0.63996	1.54412894	10	0.42988	0.40680778	10	17.1%	0.18 [-0.70, 1.06]	2020
Tang 2020	5.89	2.184239	8	6.33	4.12615656	7	13.2%	-0.13 [-1.14, 0.89]	2020
**Total (95% CI)**			**68**			**174**	**100.0%**	**0.44 [0.05, 0.83]**	

Heterogeneity: Tau² = 0.03; Chi² = 2.44, df = 2 (P = 0.30); I² = 18%
Test for overall effect: Z = 2.21 (P = 0.03)

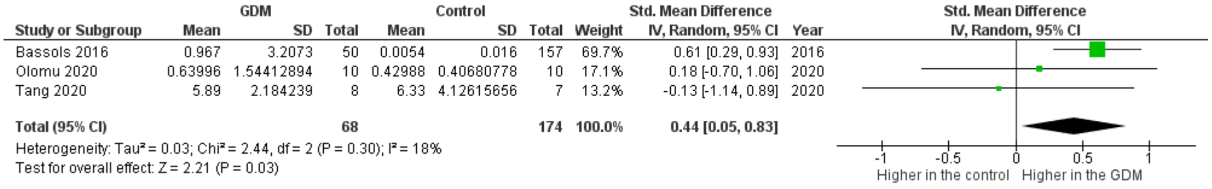

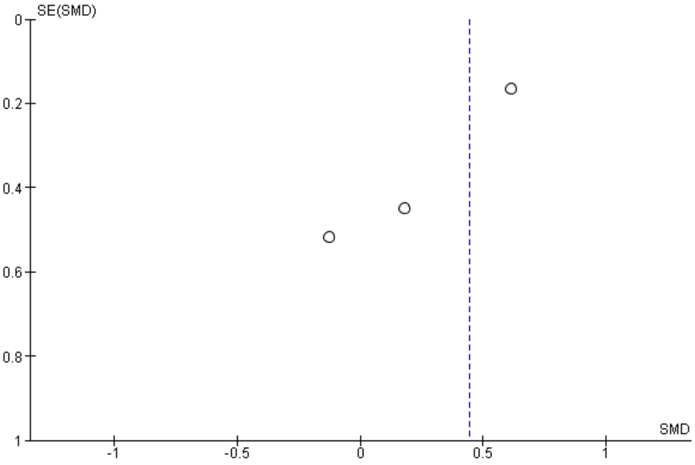

Bacteroides

Study or Subgroup	GDM Mean	GDM SD	GDM Total	Control Mean	Control SD	Control Total	Weight	Std. Mean Difference IV, Random, 95% CI	Year
Bassols 2016	1.7275	4.1665	50	0.5706	1.1681	157	80.8%	0.51 [0.18, 0.83]	2016
Olomu 2020	0.6547	0.54875567	10	0.7377	1.68232191	10	11.0%	-0.06 [-0.94, 0.81]	2020
Tang 2020	7.85	4.98717756	8	7.62	9.71673031	7	8.2%	0.03 [-0.99, 1.04]	2020
**Total (95% CI)**			**68**			**174**	**100.0%**	**0.40 [0.11, 0.70]**	

Heterogeneity: Tau² = 0.00; Chi² = 2.00, df = 2 (P = 0.37); I² = 0%
Test for overall effect: Z = 2.72 (P = 0.006)

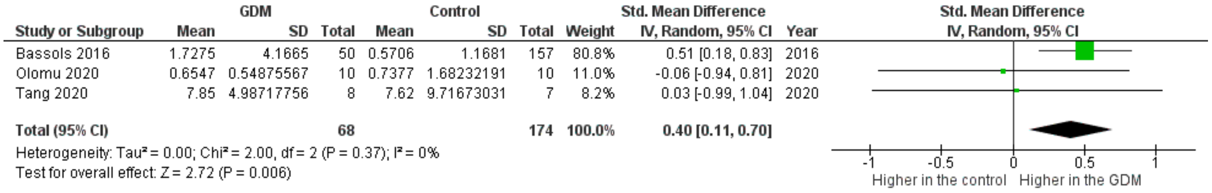

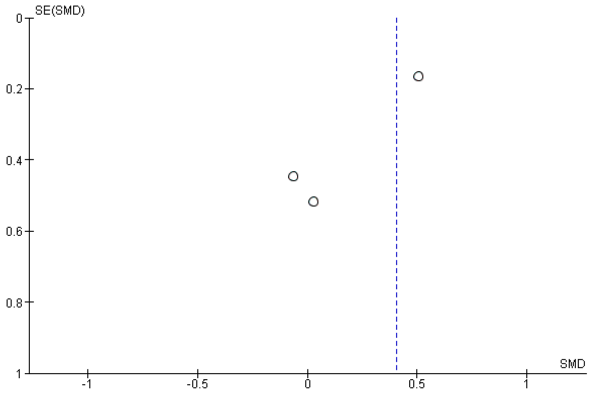

Bifidobacterium

Study or Subgroup	GDM Mean	GDM SD	GDM Total	Control Mean	Control SD	Control Total	Weight	Std. Mean Difference IV, Random, 95% CI	Year
Bassols 2016	0.1948	0.6279	50	0.0057	0.0099	157	50.5%	0.61 [0.29, 0.94]	2016
Olomu 2020	0.4115896	0.32533178	10	1.9004	8.56631015	10	26.8%	-0.24 [-1.12, 0.65]	2020
Tang 2020	4.16	1.4889137	8	4.39	1.99047105	7	22.7%	-0.12 [-1.14, 0.89]	2020
**Total (95% CI)**			**68**			**174**	**100.0%**	**0.22 [-0.40, 0.84]**	

Heterogeneity: Tau² = 0.17; Chi² = 4.53, df = 2 (P = 0.10); I² = 56%
Test for overall effect: Z = 0.69 (P = 0.49)

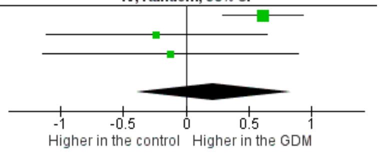

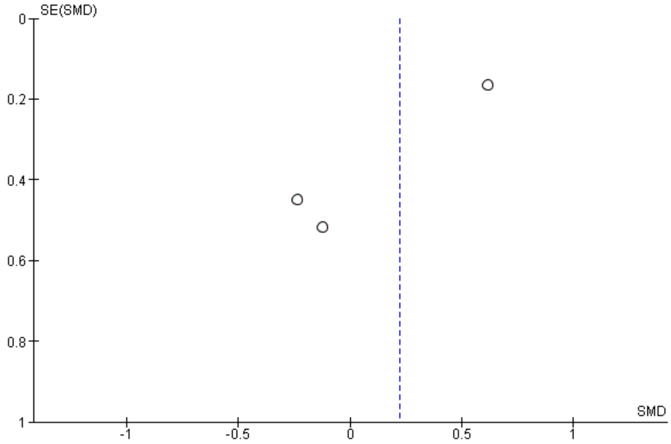

Blautia

Study or Subgroup	GDM Mean	GDM SD	GDM Total	Control Mean	Control SD	Control Total	Weight	Std. Mean Difference IV, Random, 95% CI	Year
Bassols 2016	0.1294	0.4294	50	0.0738	0.2408	157	81.3%	0.19 [-0.13, 0.51]	2016
Olomu 2020	0.2889	0.40796715	10	0.36765	0.86845868	10	10.7%	-0.11 [-0.99, 0.77]	2020
Tang 2020	8.33	7.04226441	8	7.9	3.3447257	7	8.0%	0.07 [-0.94, 1.09]	2020
**Total (95% CI)**			**68**			**174**	**100.0%**	**0.15 [-0.14, 0.43]**	

Heterogeneity: Tau² = 0.00; Chi² = 0.41, df = 2 (P = 0.81); I² = 0%
Test for overall effect: Z = 0.99 (P = 0.32)

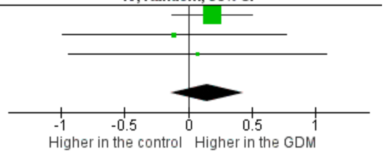

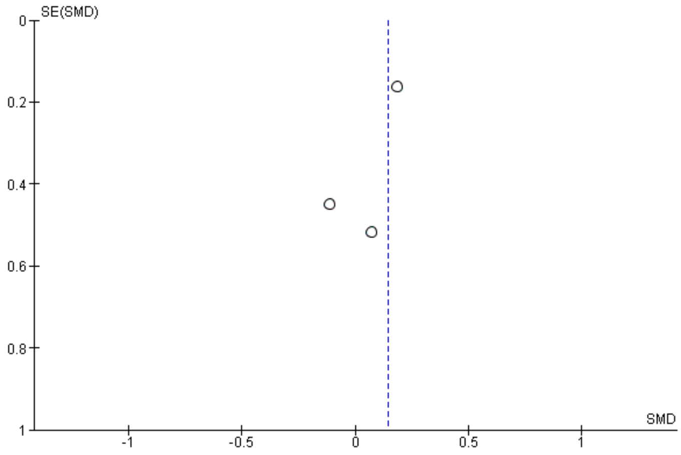

Lachnospiraceae uncl.

Study or Subgroup	GDM Mean	GDM SD	GDM Total	Control Mean	Control SD	Control Total	Weight	Std. Mean Difference IV, Random, 95% CI	Year
Bassols 2016	0.2549	0.8407	50	0.0827	0.269	157	81.2%	0.36 [0.04, 0.68]	2016
Olomu 2020	0.28618	0.50910962	10	0.2453	0.6769771	10	10.8%	0.07 [-0.81, 0.94]	2020
Tang 2020	2.57	0.7374361	8	2.34	1.22376468	7	8.0%	0.22 [-0.80, 1.24]	2020
**Total (95% CI)**			**68**			**174**	**100.0%**	**0.32 [0.03, 0.61]**	

Heterogeneity: Tau² = 0.00; Chi² = 0.43, df = 2 (P = 0.81); I² = 0%
Test for overall effect: Z = 2.17 (P = 0.03)

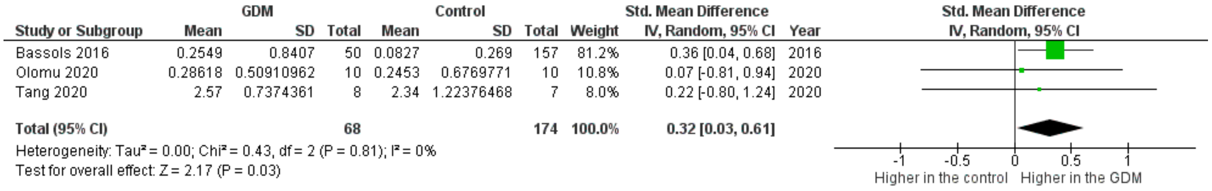

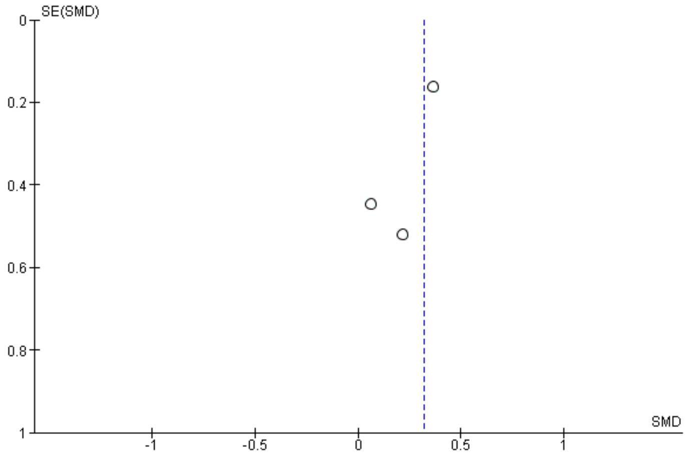

Gut

Phylum

Actinobacteria

All

Study or Subgroup	GDM Mean	GDM SD	GDM Total	Control Mean	Control SD	Control Total	Weight	Std. Mean Difference IV, Random, 95% CI
1.st T, Zheng 2020	2.1576	2.7539	31	2.249	3.9281	103	9.5%	-0.02 [-0.43, 0.38]
1st/2nd T, Hu 2021	4.53	0.06	201	6.94	0.08	201	7.5%	-34.02 [-36.39, -31.65]
2.nd T, Zhang X 2021	3.88792952	1.72733333	7	3.88792952	2.28758519	27	9.2%	0.00 [-0.83, 0.83]
2.nd T, Zhang Y 2021	4.9	7	128	6.1	7.4	709	9.5%	-0.16 [-0.35, 0.03]
2.nd T, Zheng 2020	2.8213	3.1744	31	3.2512	36,078	103	9.5%	-0.00 [-0.40, 0.40]
2nd/3rd T, Su 2021	2	1.55555556	27	26	1.7037037	71	7.9%	-14.30 [-16.39, -12.21]
2nd/3rd T, Wei 2022	3.339	3.145	15	3.629	2.614	18	9.3%	-0.10 [-0.78, 0.59]
2nd/3rd T, Wu N 2021	4.9091	10.633	27	4.8788	9.0798	30	9.4%	0.00 [-0.52, 0.52]
3rd T, Benítez-Guerrero 2022	14.35	4.065355	11	12.22	1.115686	30	9.3%	0.92 [0.20, 1.64]
3rd T, Wang J 2018	4.88	3.216	74	5.795	6.867	73	9.5%	-0.17 [-0.49, 0.15]
3rd T, Wu Y 2020	15.796	16.01	23	5.182	4.511	26	9.4%	0.91 [0.32, 1.51]
**Total (95% CI)**			**575**			**1391**	**100.0%**	**-3.55 [-4.96, -2.14]**

Heterogeneity: Tau² = 5.42; Chi² = 981.64, df = 10 (P < 0.00001); I² = 99%
Test for overall effect: Z = 4.94 (P < 0.00001)

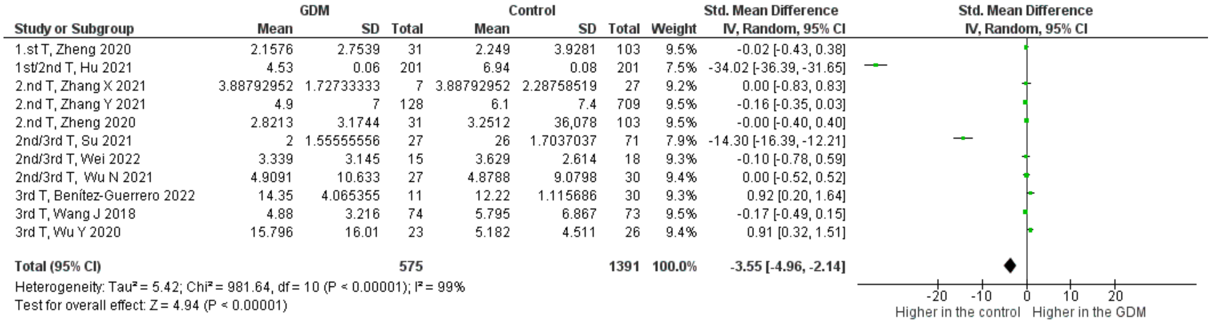

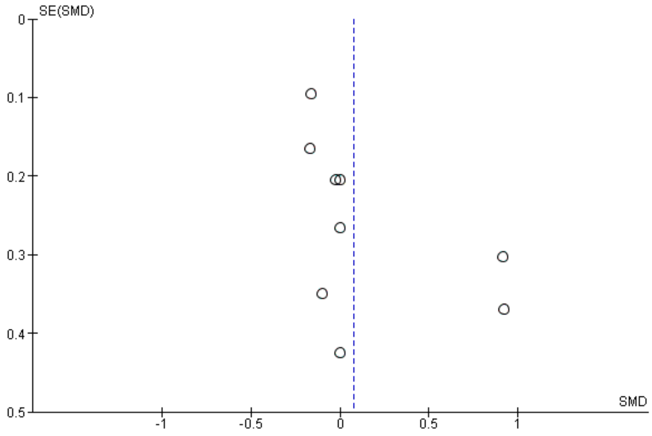

Without outliers

Study or Subgroup	GDM Mean	GDM SD	GDM Total	Control Mean	Control SD	Control Total	Weight	Std. Mean Difference IV, Random, 95% CI
1.st T, Zheng 2020	2.1576	2.7539	31	2.249	3.9281	103	13.1%	-0.02 [-0.43, 0.38]
1st/2nd T, Hu 2021	4.53	0.06	201	6.94	0.08	201	0.0%	-34.02 [-36.39, -31.65]
2.nd T, Zhang X 2021	3.88792952	1.72733333	7	3.88792952	2.28758519	27	5.7%	0.00 [-0.83, 0.83]
2.nd T, Zhang Y 2021	4.9	7	128	6.1	7.4	709	19.1%	-0.16 [-0.35, 0.03]
2.nd T, Zheng 2020	2.8213	3.1744	31	3.2512	36,078	103	13.1%	-0.00 [-0.40, 0.40]
2nd/3rd T, Su 2021	2	1.55555556	27	26	1.7037037	71	0.0%	-14.30 [-16.39, -12.21]
2nd/3rd T, Wei 2022	3.339	3.145	15	3.629	2.614	18	7.4%	-0.10 [-0.78, 0.59]
2nd/3rd T, Wu N 2021	4.9091	10.633	27	4.8788	9.0798	30	10.3%	0.00 [-0.52, 0.52]
3rd T, Benítez-Guerrero 2022	14.35	4.065355	11	12.22	1.115686	30	6.9%	0.92 [0.20, 1.64]
3rd T, Wang J 2018	4.88	3.216	74	5.795	6.867	73	15.3%	-0.17 [-0.49, 0.15]
3rd T, Wu Y 2020	15.796	16.01	23	5.182	4.511	26	8.9%	0.91 [0.32, 1.51]
**Total (95% CI)**			**347**			**1119**	**100.0%**	**0.08 [-0.15, 0.31]**

Heterogeneity: Tau² = 0.06; Chi² = 19.11, df = 8 (P = 0.01); I² = 58%
Test for overall effect: Z = 0.66 (P = 0.51)

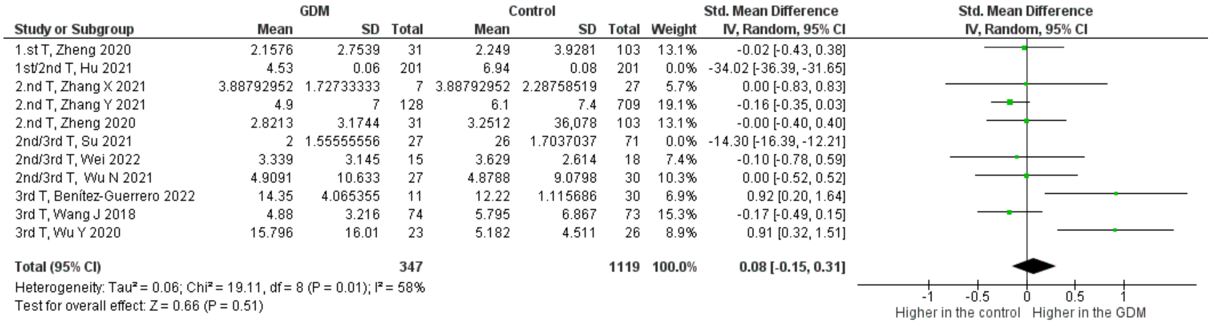

Bacteroidetes

All

Study or Subgroup	GDM Mean	GDM SD	GDM Total	Control Mean	Control SD	Control Total	Weight	Std. Mean Difference IV, Random, 95% CI
1.st T, Zheng 2020	50.3486	14.3169	31	51.2508	15.4794	103	9.4%	-0.06 [-0.46, 0.34]
1st/2nd T, Hu 2021	15.41	0.15	201	13.16	0.14	201	8.9%	15.48 [14.39, 16.57]
2.nd T, Zhang X 2021	37.57493956	12.16007407	7	34.9335	10.91814815	27	9.1%	0.23 [-0.60, 1.06]
2.nd T, Zhang Y 2021	17.7	15.6	128	14.6	13	709	9.5%	0.23 [0.04, 0.42]
2.nd T, Zheng 2020	44.3409	18.5185	31	44.7108	3.8743	103	9.4%	-0.04 [-0.44, 0.36]
2nd/3rd T, Su 2021	30	18	27	31	20	71	9.4%	-0.05 [-0.49, 0.39]
2nd/3rd T, Wei 2022	38.493	14.629	15	49.312	8.616	18	9.2%	-0.90 [-1.62, -0.18]
2nd/3rd T, Wu N 2021	35.7108	27.6579	27	35.3704	23.5618	30	9.4%	0.01 [-0.51, 0.53]
3rd T, Benítez-Guerrero 2022	0	0	11	3.38	0.376194	30	7.0%	-10.22 [-12.64, -7.79]
3rd T, Wang J 2018	33.73	9.03	74	30.735	14.127	73	9.4%	0.25 [-0.07, 0.58]
3rd T, Wu Y 2020	55.594	24.336	23	55.973	21.5	26	9.3%	-0.02 [-0.58, 0.54]
**Total (95% CI)**			**575**			**1391**	**100.0%**	**0.63 [-0.61, 1.87]**

Heterogeneity: Tau² = 4.21; Chi² = 834.80, df = 10 (P < 0.00001); I² = 99%
Test for overall effect: Z = 0.99 (P = 0.32)

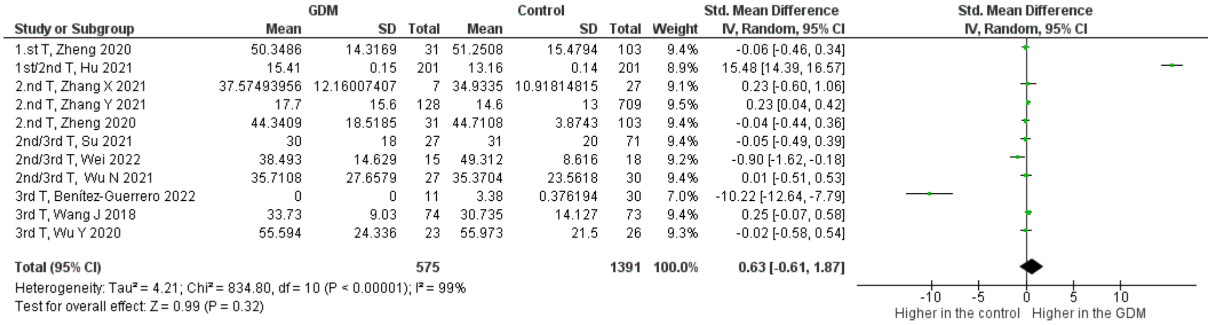

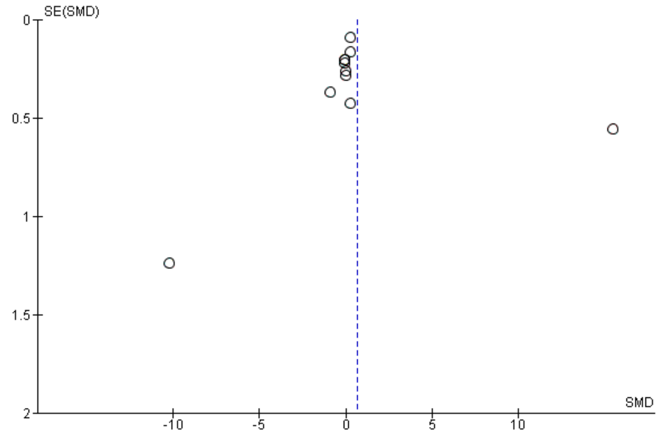

Without outliers

Study or Subgroup	GDM Mean	GDM SD	GDM Total	Control Mean	Control SD	Control Total	Weight	Std. Mean Difference IV, Random, 95% CI
1.st T, Zheng 2020	50.3486	14.3169	31	51.2508	15.4794	103	12.1%	-0.06 [-0.46, 0.34]
1st/2nd T, Hu 2021	15.41	0.15	201	13.16	0.14	201	0.0%	15.48 [14.39, 16.57]
2.nd T, Zhang X 2021	37.57493956	12.16007407	7	34.9335	10.91814815	27	3.7%	0.23 [-0.60, 1.06]
2.nd T, Zhang Y 2021	17.7	15.6	128	14.6	13	709	25.4%	0.23 [0.04, 0.42]
2.nd T, Zheng 2020	44.3409	18.5185	31	44.7108	3.8743	103	12.1%	-0.04 [-0.44, 0.36]
2nd/3rd T, Su 2021	30	18	27	31	20	71	10.5%	-0.05 [-0.49, 0.39]
2nd/3rd T, Wei 2022	38.493	14.629	15	49.312	8.616	18	4.8%	-0.90 [-1.62, -0.18]
2nd/3rd T, Wu N 2021	35.7108	27.6579	27	35.3704	23.5618	30	8.3%	0.01 [-0.51, 0.53]
3rd T, Benítez-Guerrero 2022	0	0	11	3.38	0.376194	30	0.0%	-10.22 [-12.64, -7.79]
3rd T, Wang J 2018	33.73	9.03	74	30.735	14.127	73	15.7%	0.25 [-0.07, 0.58]
3rd T, Wu Y 2020	55.594	24.336	23	55.973	21.5	26	7.4%	-0.02 [-0.58, 0.54]
**Total (95% CI)**			**363**			**1160**	**100.0%**	**0.05 [-0.12, 0.22]**

Heterogeneity: $Tau^2 = 0.02$; $Chi^2 = 11.91$, df = 8 (P = 0.16); $I^2 = 33\%$
Test for overall effect: Z = 0.53 (P = 0.59)

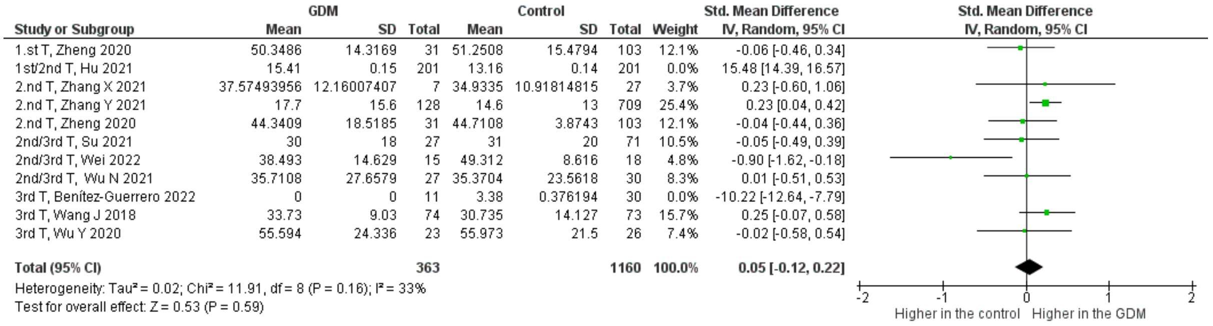

Firmicutes

All

Study or Subgroup	GDM Mean	GDM SD	GDM Total	Control Mean	Control SD	Control Total	Weight	Std. Mean Difference IV, Random, 95% CI
1.st T, Zheng 2020	44.6353	13.5598	31	43.6481	14.7393	103	9.2%	0.07 [-0.33, 0.47]
1st/2nd T, Hu 2021	74.04	0.14	201	74.72	0.15	201	9.2%	-4.68 [-5.06, -4.30]
2.nd T, Zhang X 2021	50.1851	8.12518518	7	52.3151569	15.00422222	27	8.7%	-0.15 [-0.98, 0.68]
2.nd T, Zhang Y 2021	71.5	15.2	128	73.2	13.9	709	9.3%	-0.12 [-0.31, 0.07]
2.nd T, Zheng 2020	49.7556	17.3519	31	49.2711	161,790	103	9.2%	0.00 [-0.40, 0.40]
2nd/3rd T, Su 2021	57	19	27	54	19	71	9.2%	0.16 [-0.29, 0.60]
2nd/3rd T, Wei 2022	53.49	14.412	15	42.658	8.5	18	8.9%	0.91 [0.19, 1.64]
2nd/3rd T, Wu N 2021	54.3768	23.5192	27	54.7472	22.0243	30	9.1%	-0.02 [-0.54, 0.50]
3rd T, Benítez-Guerrero 2022	38.02	16.489274	11	21.15	6.25617	30	8.8%	1.66 [0.88, 2.45]
3rd T, Wang J 2018	54.116	21.178	74	49.87	17.889	73	9.3%	0.22 [-0.11, 0.54]
3rd T, Wu Y 2020	30.303	16.013	23	32.513	19.868	26	9.1%	-0.12 [-0.68, 0.44]
**Total (95% CI)**			**575**			**1391**	**100.0%**	**-0.20 [-1.14, 0.74]**

Heterogeneity: $Tau^2 = 2.44$; $Chi^2 = 568.61$, df = 10 (P < 0.00001); $I^2 = 98\%$
Test for overall effect: Z = 0.42 (P = 0.68)

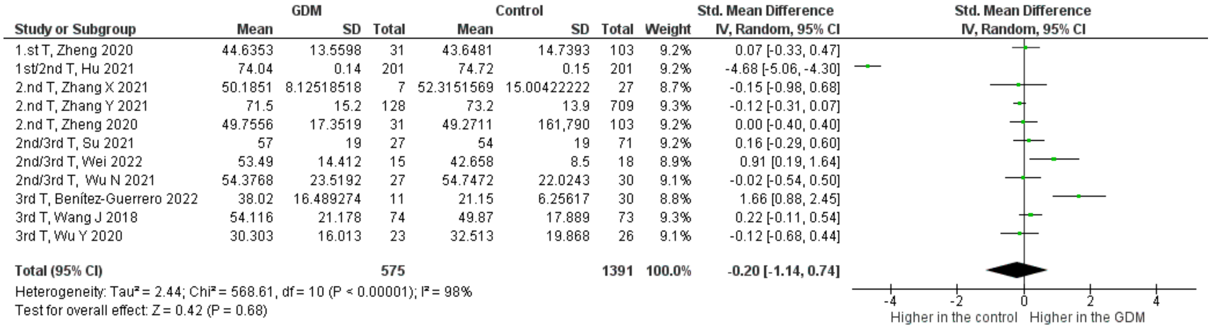

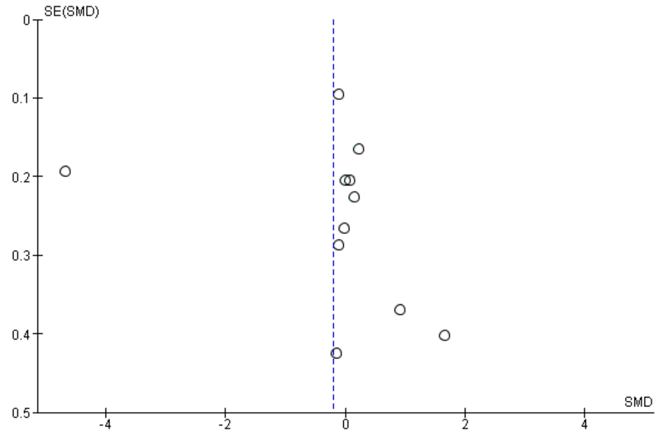

Without outlier

Study or Subgroup	GDM Mean	GDM SD	GDM Total	Control Mean	Control SD	Control Total	Weight	Std. Mean Difference IV, Random, 95% CI
1.st T, Zheng 2020	44.6353	13.5598	31	43.6481	14.7393	103	11.7%	0.07 [-0.33, 0.47]
1st/2nd T, Hu 2021	74.04	0.14	201	74.72	0.15	201	0.0%	-4.68 [-5.06, -4.30]
2.nd T, Zhang X 2021	50.1851	8.12518518	7	52.3151569	15.00422222	27	5.7%	-0.15 [-0.98, 0.68]
2.nd T, Zhang Y 2021	71.5	15.2	128	73.2	13.9	709	15.6%	-0.12 [-0.31, 0.07]
2.nd T, Zheng 2020	49.7556	17.3519	31	49.2711	161,790	103	11.7%	0.00 [-0.40, 0.40]
2nd/3rd T, Su 2021	57	19	27	54	19	71	10.9%	0.16 [-0.29, 0.60]
2nd/3rd T, Wei 2022	53.49	14.412	15	42.658	8.5	18	6.8%	0.91 [0.19, 1.64]
2nd/3rd T, Wu N 2021	54.3768	23.5192	27	54.7472	22.0243	30	9.6%	-0.02 [-0.54, 0.50]
3rd T, Benítez-Guerrero 2022	38.02	16.489274	11	21.15	6.25617	30	6.1%	1.66 [0.88, 2.45]
3rd T, Wang J 2018	54.116	21.178	74	49.87	17.889	73	13.1%	0.22 [-0.11, 0.54]
3rd T, Wu Y 2020	30.303	16.013	23	32.513	19.868	26	8.9%	-0.12 [-0.68, 0.44]
**Total (95% CI)**			**374**			**1190**	**100.0%**	**0.18 [-0.06, 0.42]**

Heterogeneity: Tau² = 0.09; Chi² = 26.66, df = 9 (P = 0.002); I² = 66%
Test for overall effect: Z = 1.43 (P = 0.15)

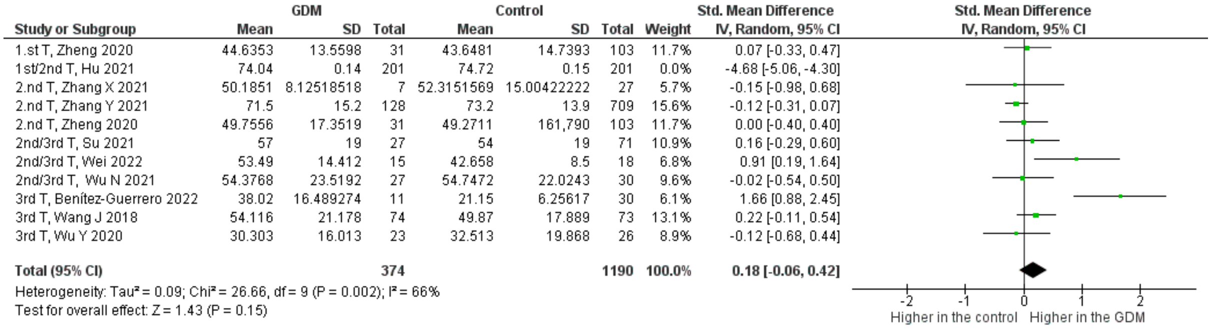

Fusobacteriota

Study or Subgroup	GDM Mean	GDM SD	GDM Total	Control Mean	Control SD	Control Total	Weight	Std. Mean Difference IV, Random, 95% CI
2.nd T, Zhang X 2021	1.19589	0.63097778	7	0.6816298	0.31553333	27	23.8%	1.27 [0.38, 2.16]
2nd/3rd T, Wu N 2021	0.0694	0.296	27	0.4895	6.5173	30	35.0%	-0.09 [-0.61, 0.43]
3rd T, Wang J 2018	3.805	3.063	74	4.092	5.217	73	41.2%	-0.07 [-0.39, 0.26]
**Total (95% CI)**			**108**			**130**	**100.0%**	**0.24 [-0.38, 0.87]**

Heterogeneity: Tau² = 0.22; Chi² = 8.00, df = 2 (P = 0.02); I² = 75%
Test for overall effect: Z = 0.77 (P = 0.44)

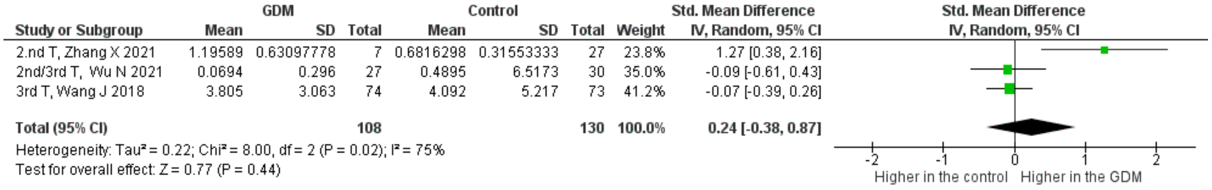

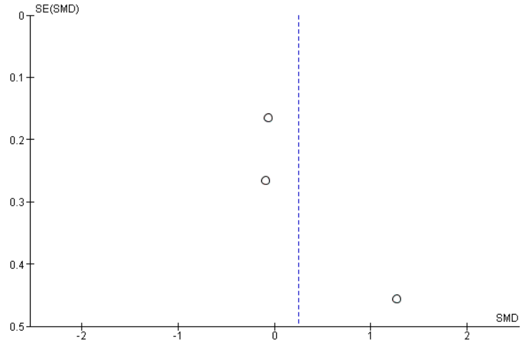

Proteobacteria

All

Study or Subgroup	GDM Mean	GDM SD	GDM Total	Control Mean	Control SD	Control Total	Weight	Std. Mean Difference IV, Random, 95% CI
1.st T, Zheng 2020	2.8893	3.4235	31	2.7255	2.0009	103	9.3%	0.07 [-0.33, 0.47]
1st/2nd T, Hu 2021	4.45	0.08	201	3.19	0.04	201	8.3%	19.88 [18.49, 21.28]
2.nd T, Zhang X 2021	5.7944798	2.14915556	7	6.0096648	3.25954296	27	8.9%	-0.07 [-0.90, 0.76]
2.nd T, Zhang Y 2021	5.7	8	128	5.5	8.3	709	9.3%	0.02 [-0.16, 0.21]
2.nd T, Zheng 2020	2.6317	1.5116	31	2.5495	2.0347	103	9.3%	0.04 [-0.36, 0.44]
2nd/3rd T, Su 2021	9	5.92592593	27	10	7.40740741	71	9.2%	-0.14 [-0.58, 0.30]
2nd/3rd T, Wei 2022	3.29	3.475	15	3.749	3.753	18	9.1%	-0.12 [-0.81, 0.56]
2nd/3rd T, Wu N 2021	2.9394	1.8059	27	2.9697	3.8922	30	9.2%	-0.01 [-0.53, 0.51]
3rd T, Benítez-Guerrero 2022	46.82	20.230922	11	62.77	20.996565	30	9.0%	-0.75 [-1.46, -0.04]
3rd T, Wang J 2018	11.266	8.313	74	9.645	12.394	73	9.3%	0.15 [-0.17, 0.48]
3rd T, Wu Y 2020	6.264	13.785	23	9.725	8.582	26	9.2%	-0.30 [-0.87, 0.26]
**Total (95% CI)**			**575**			**1391**	**100.0%**	**1.54 [0.37, 2.71]**

Heterogeneity: Tau² = 3.79; Chi² = 782.39, df = 10 (P < 0.00001); I² = 99%
Test for overall effect: Z = 2.59 (P = 0.010)

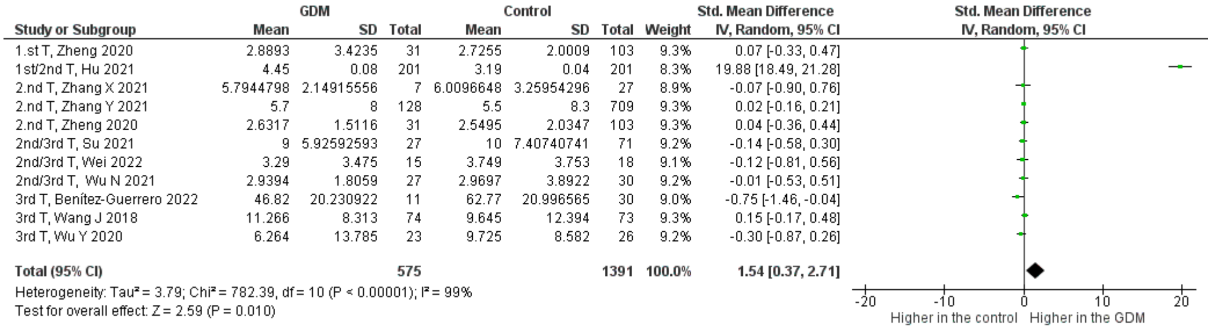

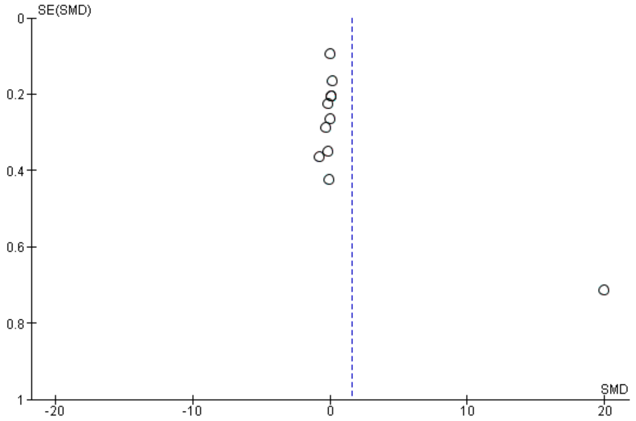

Without outlier

Study or Subgroup	GDM Mean	GDM SD	GDM Total	Control Mean	Control SD	Control Total	Weight	Std. Mean Difference IV, Random, 95% CI
1.st T, Zheng 2020	2.8893	3.4235	31	2.7255	2.0009	103	9.2%	0.07 [-0.33, 0.47]
1st/2nd T, Hu 2021	4.45	0.08	201	3.19	0.04	201	0.0%	19.88 [18.49, 21.28]
2.nd T, Zhang X 2021	5.7944798	2.14915556	7	6.0096648	3.25954296	27	2.1%	-0.07 [-0.90, 0.76]
2.nd T, Zhang Y 2021	5.7	8	128	5.5	8.3	709	41.7%	0.02 [-0.16, 0.21]
2.nd T, Zheng 2020	2.6317	1.5116	31	2.5495	2.0347	103	9.2%	0.04 [-0.36, 0.44]
2nd/3rd T, Su 2021	9	5.92592593	27	10	7.40740741	71	7.5%	-0.14 [-0.58, 0.30]
2nd/3rd T, Wei 2022	3.29	3.475	15	3.749	3.753	18	3.1%	-0.12 [-0.81, 0.56]
2nd/3rd T, Wu N 2021	2.9394	1.8059	27	2.9697	3.8922	30	5.5%	-0.01 [-0.53, 0.51]
3rd T, Benítez-Guerrero 2022	46.82	20.230922	11	62.77	20.996565	30	2.9%	-0.75 [-1.46, -0.04]
3rd T, Wang J 2018	11.266	8.313	74	9.645	12.394	73	14.1%	0.15 [-0.17, 0.48]
3rd T, Wu Y 2020	6.264	13.785	23	9.725	8.582	26	4.6%	-0.30 [-0.87, 0.26]
**Total (95% CI)**			**374**			**1190**	**100.0%**	**-0.01 [-0.13, 0.11]**

Heterogeneity: Tau² = 0.00; Chi² = 6.96, df = 9 (P = 0.64); I² = 0%
Test for overall effect: Z = 0.17 (P = 0.86)

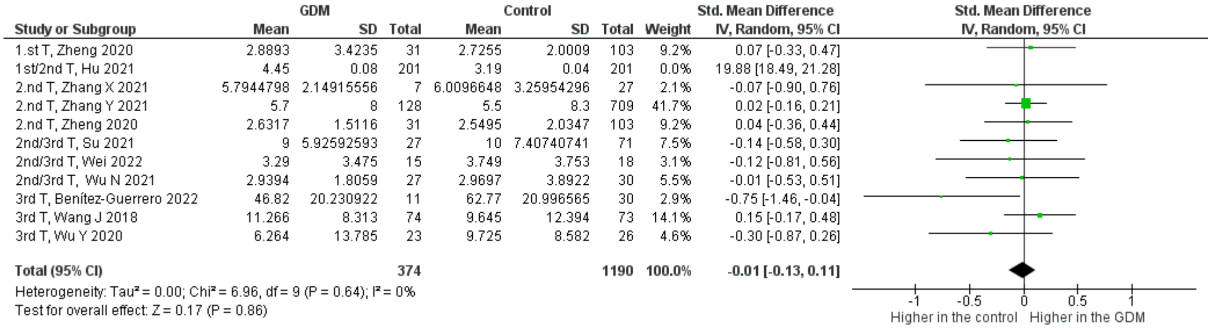

Verrucomicrobia

All

Study or Subgroup	GDM Mean	GDM SD	GDM Total	Control Mean	Control SD	Control Total	Weight	Std. Mean Difference IV, Random, 95% CI
1.st T, Zheng 2020	0.3103	0.1801	31	0.6401	0.4389	103	14.4%	-0.83 [-1.24, -0.42]
1st/2nd T, Hu 2021	4.45	0.08	201	3.19	0.04	201	13.8%	19.88 [18.49, 21.28]
2.nd T, Zheng 2020	0.7368	0.7137	31	0.7274	0.8822	103	14.4%	0.01 [-0.39, 0.41]
2nd/3rd T, Su 2021	0.7	0.4444444	27	0.9	0.14814815	71	14.4%	-0.75 [-1.21, -0.30]
2nd/3rd T, Wei 2022	2.172	1.208	15	3.521	2.373	18	14.3%	-0.68 [-1.39, 0.03]
2nd/3rd T, Wu N 2021	0.1394	0.1297	27	0.1395	0.2181	30	14.4%	-0.00 [-0.52, 0.52]
3rd T, Wu Y 2020	1.584	0.747	23	4.49	4.152	26	14.3%	-0.93 [-1.52, -0.34]
**Total (95% CI)**			**355**			**552**	**100.0%**	**2.28 [-0.07, 4.64]**

Heterogeneity: Tau² = 9.98; Chi² = 818.69, df = 6 (P < 0.00001); I² = 99%
Test for overall effect: Z = 1.90 (P = 0.06)

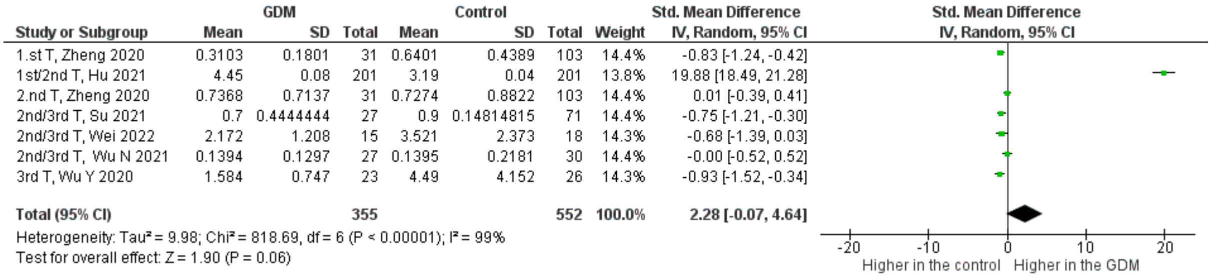

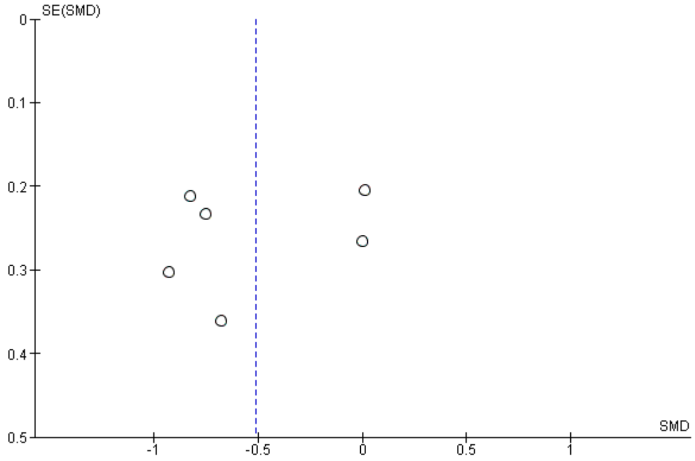

Without outlier

Study or Subgroup	GDM Mean	GDM SD	GDM Total	Control Mean	Control SD	Control Total	Weight	Std. Mean Difference IV, Random, 95% CI
1.st T, Zheng 2020	0.3103	0.1801	31	0.6401	0.4389	103	18.8%	-0.83 [-1.24, -0.42]
1st/2nd T, Hu 2021	4.45	0.08	201	3.19	0.04	201	0.0%	19.88 [18.49, 21.28]
2.nd T, Zheng 2020	0.7368	0.7137	31	0.7274	0.8822	103	19.1%	0.01 [-0.39, 0.41]
2nd/3rd T, Su 2021	0.7	0.4444444	27	0.9	0.14814815	71	17.9%	-0.75 [-1.21, -0.30]
2nd/3rd T, Wei 2022	2.172	1.208	15	3.521	2.373	18	12.7%	-0.68 [-1.39, 0.03]
2nd/3rd T, Wu N 2021	0.1394	0.1297	27	0.1395	0.2181	30	16.5%	-0.00 [-0.52, 0.52]
3rd T, Wu Y 2020	1.584	0.747	23	4.49	4.152	26	14.9%	-0.93 [-1.52, -0.34]
**Total (95% CI)**			**154**			**351**	**100.0%**	**-0.51 [-0.87, -0.15]**

Heterogeneity: Tau² = 0.13; Chi² = 15.65, df = 5 (P = 0.008); I² = 68%
Test for overall effect: Z = 2.80 (P = 0.005)

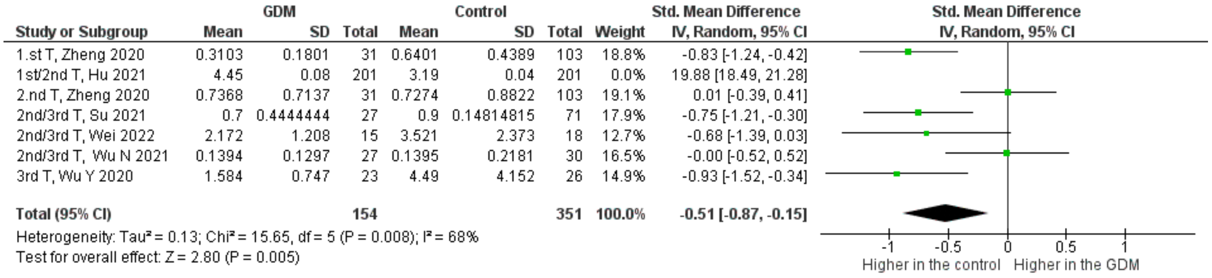

Genus

Faecalibacterium

Study or Subgroup	GDM Mean	GDM SD	GDM Total	Control Mean	Control SD	Control Total	Weight	Std. Mean Difference IV, Random, 95% CI
1.st T, Zheng 2020	9.67265821	7.6066147	31	8.57621719	5.51403374	103	16.6%	0.18 [-0.22, 0.58]
2.nd T, Zheng 2020	8.21519205	4.46386208	31	8.94387025	5.67799916	103	16.6%	-0.13 [-0.54, 0.27]
2nd/3rd T, Su 2021	18	13.33	27	16	11.85	71	14.6%	0.16 [-0.28, 0.61]
2nd/3rd T, Wang X 2020	0.00934	0.0151	59	0.0136	0.0159	48	17.6%	-0.27 [-0.66, 0.11]
2nd/3rd T, Xu 2020	19.472	14.381	43	20.77	12.406	81	18.3%	-0.10 [-0.47, 0.27]
2nd/3rd T, Ye 2019	0.044	0.055	12	0.136	0.106	16	5.8%	-1.01 [-1.82, -0.21]
3rd T, Wu Y 2020	6.415	4.914	23	7.032	6.304	26	10.4%	-0.11 [-0.67, 0.45]
**Total (95% CI)**			**226**			**448**	**100.0%**	**-0.11 [-0.31, 0.10]**

Heterogeneity: Tau² = 0.03; Chi² = 9.01, df = 6 (P = 0.17); I² = 33%
Test for overall effect: Z = 0.99 (P = 0.32)

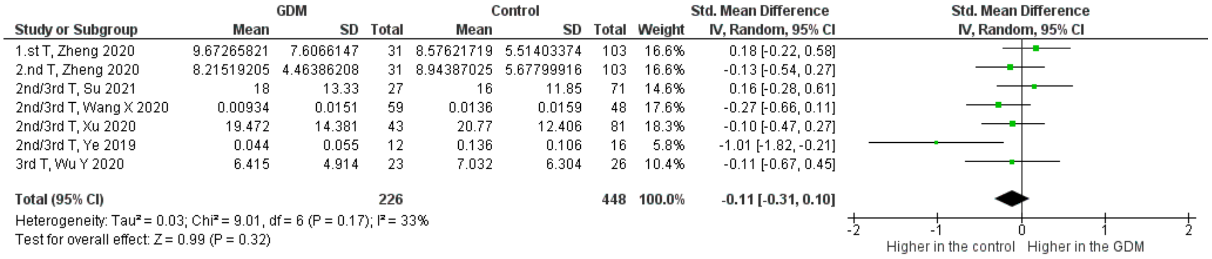

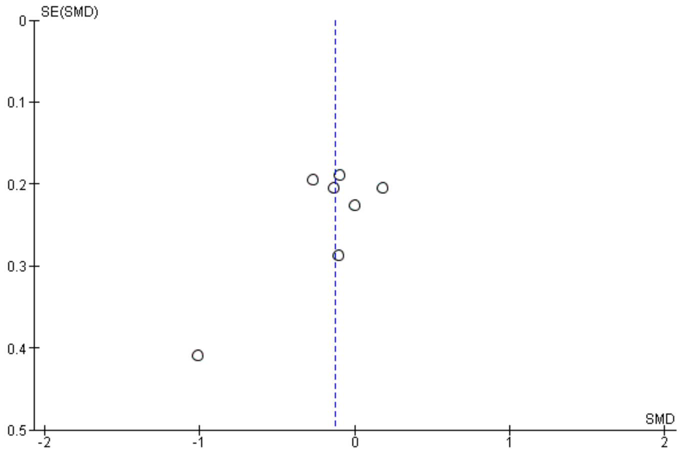

Prevotella

Study or Subgroup	GDM Mean	GDM SD	GDM Total	Control Mean	Control SD	Control Total	Weight	Std. Mean Difference IV, Random, 95% CI
3rd T, Wu Y 2020	15.421	22.59	23	19.183	20.952	26	14.1%	-0.17 [-0.73, 0.39]
1.st T, Zheng 2020	39.51119431	32.58944162	31	34.52752875	28.89987096	103	27.6%	0.17 [-0.24, 0.57]
2.nd T, Zheng 2020	31.88467857	36.64772316	31	31.4805867	28.01890048	103	27.7%	0.01 [-0.39, 0.41]
2nd/3rd T, Wang X 2020	0.000627	0.00478	59	0	0	48	30.6%	0.18 [-0.21, 0.56]
**Total (95% CI)**			**144**			**280**	**100.0%**	**0.08 [-0.13, 0.29]**

Heterogeneity: Tau² = 0.00; Chi² = 1.28, df = 3 (P = 0.73); I² = 0%
Test for overall effect: Z = 0.74 (P = 0.46)

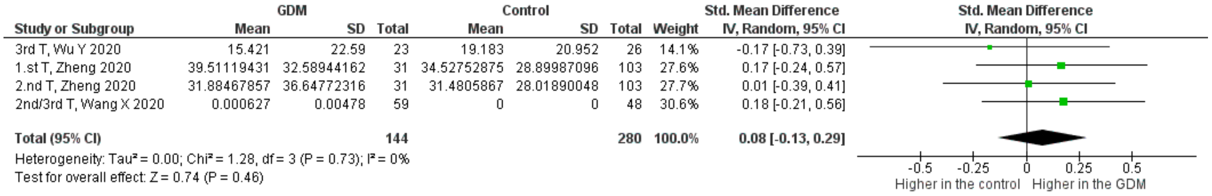

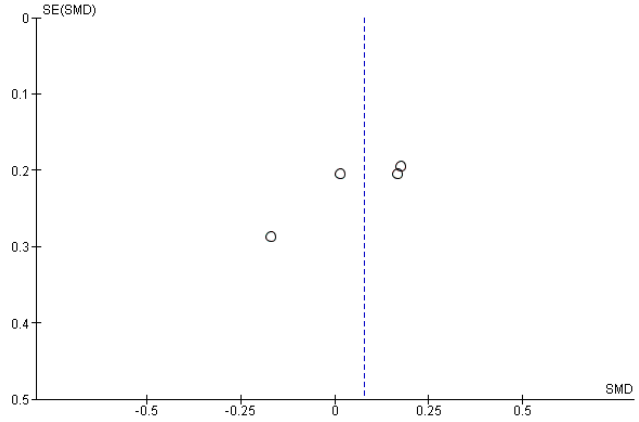

Bacteroides

Study or Subgroup	GDM Mean	GDM SD	GDM Total	Control Mean	Control SD	Control Total	Weight	Std. Mean Difference IV, Random, 95% CI
1.st T, Zheng 2020	39.9315882	17.4523818	31	39.06144101	39.06144101	103	20.7%	0.02 [-0.38, 0.43]
2.nd T, Zheng 2020	34.42253789	18.8779719	31	31.30818226	19.11143981	103	20.6%	0.16 [-0.24, 0.56]
2nd/3rd T, Su 2021	25	2,607,407,407	27	24	2,451,851,852	71	17.0%	0.00 [-0.44, 0.44]
2nd/3rd T, Xu 2020	10.244	12,465	43	8.364	10.936	81	24.4%	0.00 [-0.37, 0.37]
3rd T, Benítez-Guerrero 2022	0	0	11	2.98	11.75	30	6.9%	-0.29 [-0.98, 0.41]
3rd T, Wu Y 2020	59.612	28.869	23	53.018	28.553	26	10.5%	0.23 [-0.34, 0.79]
**Total (95% CI)**			**166**			**414**	**100.0%**	**0.04 [-0.14, 0.22]**

Heterogeneity: Tau² = 0.00; Chi² = 1.72, df = 5 (P = 0.89); I² = 0%
Test for overall effect: Z = 0.46 (P = 0.65)

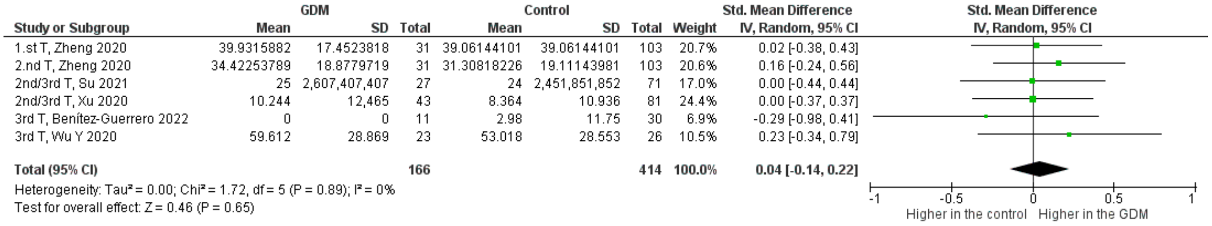

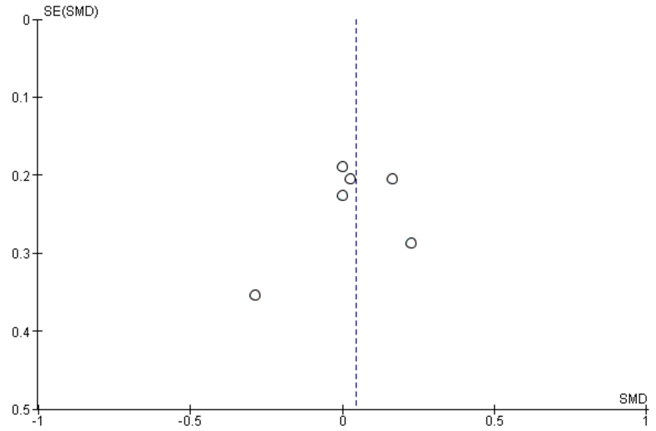

Megamonas

All

	GDM			Control				Std. Mean Difference
Study or Subgroup	Mean	SD	Total	Mean	SD	Total	Weight	IV, Random, 95% CI
1.st T, Zheng 2020	4.54277286	2.35228444	31	4.33380921	6.56845582	103	26.9%	0.04 [-0.37, 0.44]
2.nd T, Zheng 2020	3.50186634	4.30393405	31	4.37777259	10.86493646	103	26.9%	-0.09 [-0.49, 0.31]
2nd/3rd T, Su 2021	53	6	27	1.8	0.2222222	71	19.2%	16.24 [13.88, 18.60]
2nd/3rd T, Xu 2020	11.254	13.19	43	10.667	10.757	81	27.0%	0.05 [-0.32, 0.42]
**Total (95% CI)**			**132**			**358**	**100.0%**	**3.11 [1.21, 5.02]**

Heterogeneity: Tau² = 3.46; Chi² = 180.12, df = 3 (P < 0.00001); I² = 98%
Test for overall effect: Z = 3.21 (P = 0.001)

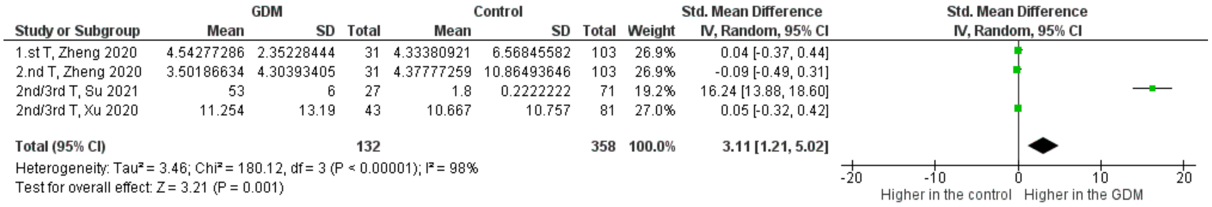

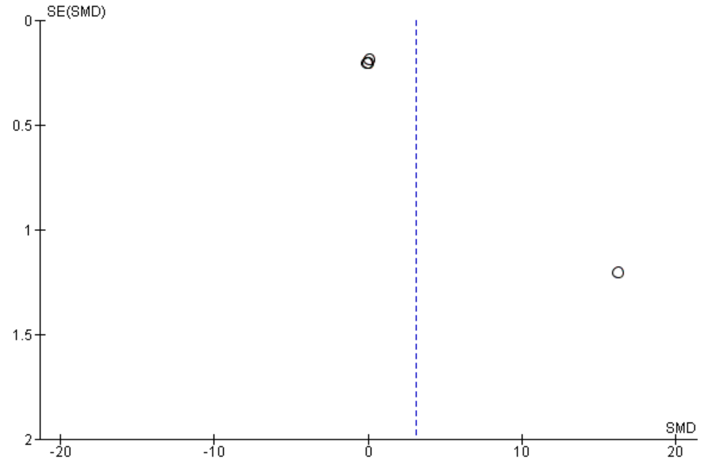

Without outlier

	GDM			Control				Std. Mean Difference
Study or Subgroup	Mean	SD	Total	Mean	SD	Total	Weight	IV, Random, 95% CI
1.st T, Zheng 2020	4.54277286	2.35228444	31	4.33380921	6.56845582	103	31.5%	0.04 [-0.37, 0.44]
2.nd T, Zheng 2020	3.50186634	4.30393405	31	4.37777259	10.86493646	103	31.4%	-0.09 [-0.49, 0.31]
2nd/3rd T, Su 2021	53	6	27	1.8	0.2222222	71	0.0%	16.24 [13.88, 18.60]
2nd/3rd T, Xu 2020	11.254	13.19	43	10.667	10.757	81	37.1%	0.05 [-0.32, 0.42]
**Total (95% CI)**			**105**			**287**	**100.0%**	**0.00 [-0.22, 0.23]**

Heterogeneity: Tau² = 0.00; Chi² = 0.29, df = 2 (P = 0.87); I² = 0%
Test for overall effect: Z = 0.01 (P = 0.99)

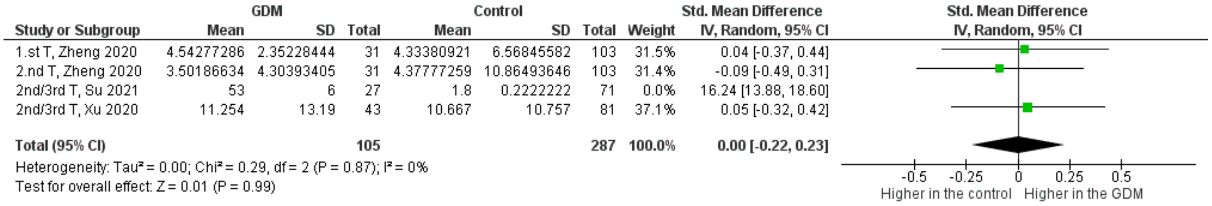

Bifidobacterium

All

Study or Subgroup	GDM Mean	GDM SD	GDM Total	Control Mean	Control SD	Control Total	Weight	Std. Mean Difference IV, Random, 95% CI
1.st T, Zheng 2020	1.49668828	2.34326503	31	1.62696531	2.11318794	103	18.4%	-0.06 [-0.46, 0.34]
2.nd T, Zheng 2020	2.19941499	2.37544897	31	2.85595625	3.68485757	103	18.4%	-0.19 [-0.59, 0.21]
2nd/3rd T, Su 2021	1.2	0.66666667	27	1.5	0.81481481	71	17.4%	-0.38 [-0.83, 0.06]
2nd/3rd T, Xu 2020	5.926	5.385	43	9.86	8.394	81	18.9%	-0.52 [-0.90, -0.15]
3rd T, Benítez-Guerrero 2022	0	0	11	1.67	4.15	30	12.5%	-0.46 [-1.16, 0.24]
3rd T, Wu Y 2020	16.693	18.255	23	5.034	4.367	26	14.4%	0.89 [0.30, 1.48]
**Total (95% CI)**			**166**			**414**	**100.0%**	**-0.14 [-0.50, 0.22]**

Heterogeneity: Tau² = 0.14; Chi² = 17.53, df = 5 (P = 0.004); I² = 71%
Test for overall effect: Z = 0.77 (P = 0.44)

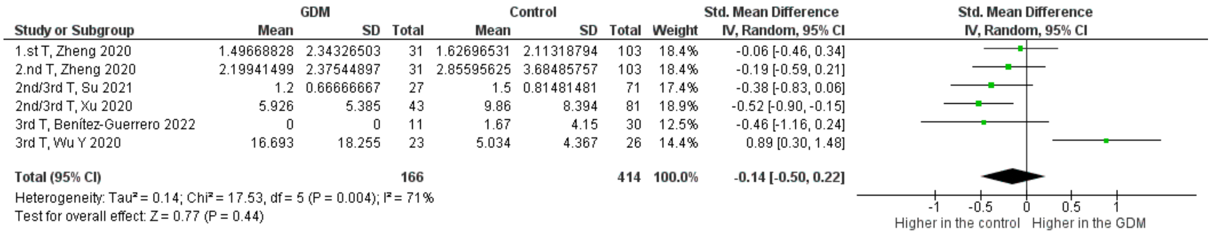

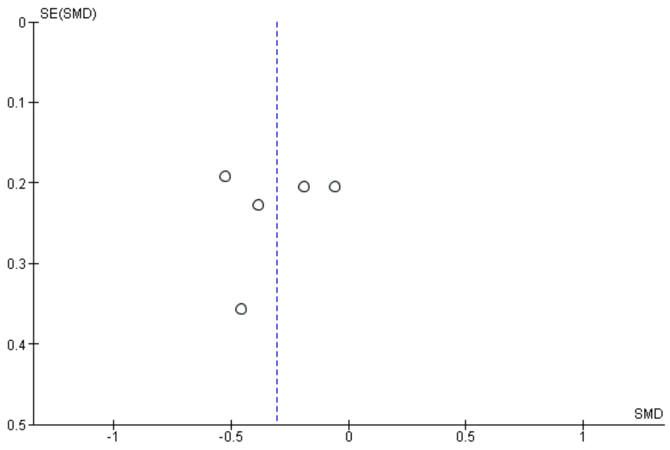

Without outlier

Study or Subgroup	GDM Mean	GDM SD	GDM Total	Control Mean	Control SD	Control Total	Weight	Std. Mean Difference IV, Random, 95% CI
1.st T, Zheng 2020	1.49668828	2.34326503	31	1.62696531	2.11318794	103	23.4%	-0.06 [-0.46, 0.34]
2.nd T, Zheng 2020	2.19941499	2.37544897	31	2.85595625	3.68485757	103	23.3%	-0.19 [-0.59, 0.21]
2nd/3rd T, Su 2021	1.2	0.66666667	27	1.5	0.81481481	71	18.9%	-0.38 [-0.83, 0.06]
2nd/3rd T, Xu 2020	5.926	5.385	43	9.86	8.394	81	26.7%	-0.52 [-0.90, -0.15]
3rd T, Benítez-Guerrero 2022	0	0	11	1.67	4.15	30	7.7%	-0.46 [-1.16, 0.24]
3rd T, Wu Y 2020	16.693	18.255	23	5.034	4.367	26	0.0%	0.89 [0.30, 1.48]
**Total (95% CI)**			**143**			**388**	**100.0%**	**-0.31 [-0.50, -0.11]**

Heterogeneity: Tau² = 0.00; Chi² = 3.32, df = 4 (P = 0.51); I² = 0%
Test for overall effect: Z = 3.08 (P = 0.002)

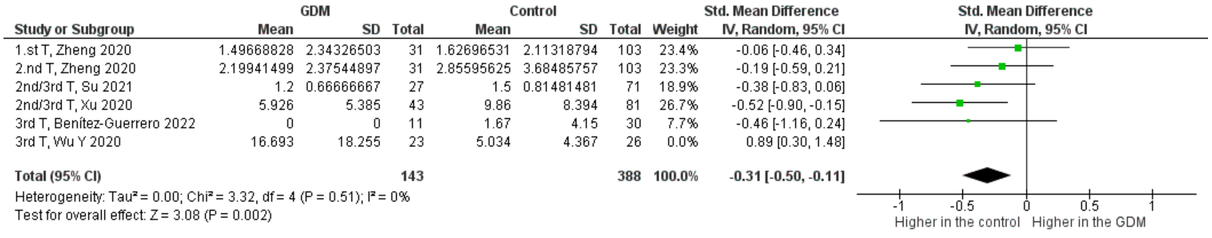

Escherichia Shigella

Study or Subgroup	GDM Mean	GDM SD	GDM Total	Control Mean	Control SD	Control Total	Weight	Std. Mean Difference IV, Random, 95% CI
2nd/3rd T, Su 2021	0.9	0.5925925	27	2.5	0.2222222	71	25.1%	-4.38 [-5.15, -3.62]
2nd/3rd T, Xu 2020	10.426	8.417	43	5.046	4.49	81	25.5%	0.87 [0.49, 1.26]
3rd T, Benítez-Guerrero 2022	8.35	2.006505	11	3.26	0.162348	30	24.1%	4.87 [3.56, 6.17]
3rd T, Wu Y 2020	9.979	19.319	23	12.172	10.684	26	25.3%	-0.14 [-0.70, 0.42]
**Total (95% CI)**			**104**			**208**	**100.0%**	**0.26 [-2.35, 2.87]**

Heterogeneity: Tau² = 6.90; Chi² = 198.39, df = 3 (P < 0.00001); I² = 98%
Test for overall effect: Z = 0.20 (P = 0.85)

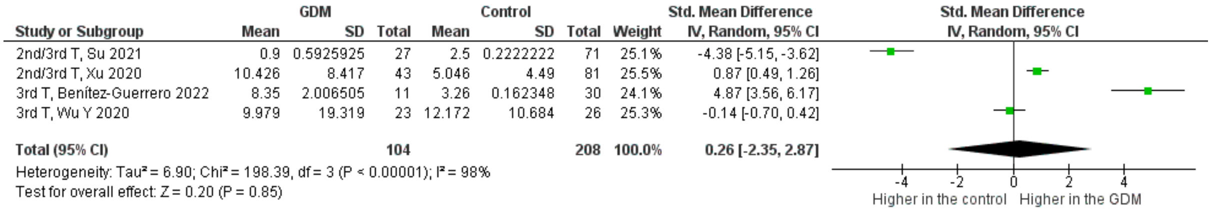

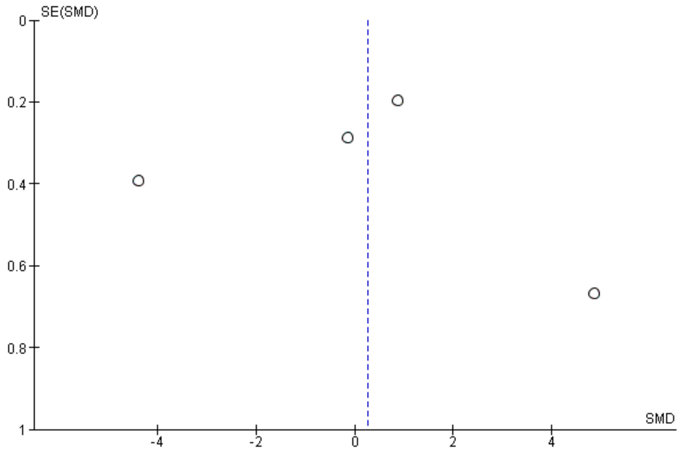

Roseburia

All

Study or Subgroup	GDM Mean	GDM SD	GDM Total	Control Mean	Control SD	Control Total	Weight	Std. Mean Difference IV, Random, 95% CI
1.st T, Zheng 2020	6.70815881	7.00507837	31	4.56370283	3.84158651	103	22.8%	0.45 [0.04, 0.85]
2.nd T, Zheng 2020	6.8807275	7.12970386	31	6.07166075	6.61304651	103	22.9%	0.12 [-0.28, 0.52]
2nd/3rd T, Wang X 2020	0.0055	0.00725	59	0.00351	0.00751	48	23.4%	0.27 [-0.11, 0.65]
2nd/3rd T, Ye 2019	0.006	0.009	12	0.024	0.019	16	12.7%	-1.12 [-1.93, -0.31]
3rd T, Wu Y 2020	5.97	4.89	23	4.27	3.478	26	18.2%	0.40 [-0.17, 0.97]
**Total (95% CI)**			**156**			**296**	**100.0%**	**0.12 [-0.26, 0.50]**

Heterogeneity: Tau² = 0.12; Chi² = 12.36, df = 4 (P = 0.01); I² = 68%
Test for overall effect: Z = 0.64 (P = 0.53)

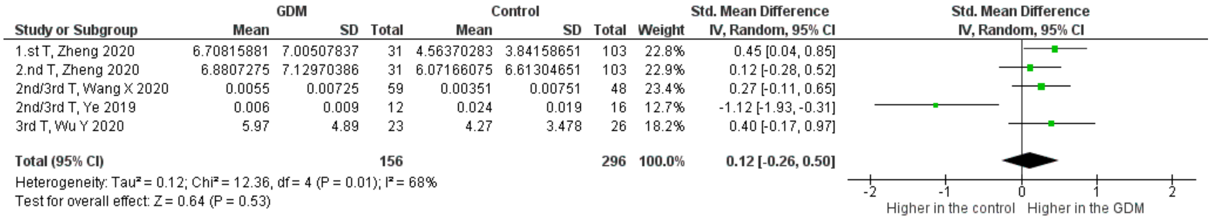

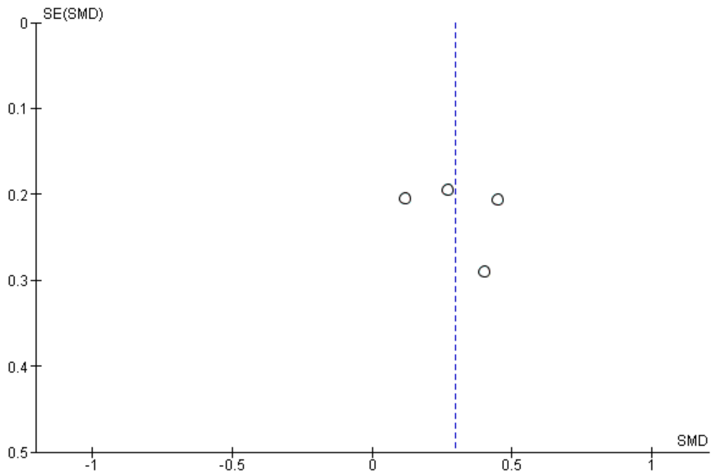

Without outlier

Study or Subgroup	GDM Mean	GDM SD	GDM Total	Control Mean	Control SD	Control Total	Weight	Std. Mean Difference IV, Random, 95% CI
1.st T, Zheng 2020	6.70815881	7.00507837	31	4.56370283	3.84158651	103	27.4%	0.45 [0.04, 0.85]
2.nd T, Zheng 2020	6.8807275	7.12970386	31	6.07166075	6.61304651	103	27.9%	0.12 [-0.28, 0.52]
2nd/3rd T, Wang X 2020	0.0055	0.00725	59	0.00351	0.00751	48	30.7%	0.27 [-0.11, 0.65]
2nd/3rd T, Ye 2019	0.006	0.009	12	0.024	0.019	16	0.0%	-1.12 [-1.93, -0.31]
3rd T, Wu Y 2020	5.97	4.89	23	4.27	3.478	26	14.0%	0.40 [-0.17, 0.97]
**Total (95% CI)**			**144**			**280**	**100.0%**	**0.29 [0.08, 0.51]**

Heterogeneity: Tau² = 0.00; Chi² = 1.43, df = 3 (P = 0.70); I² = 0%
Test for overall effect: Z = 2.72 (P = 0.007)

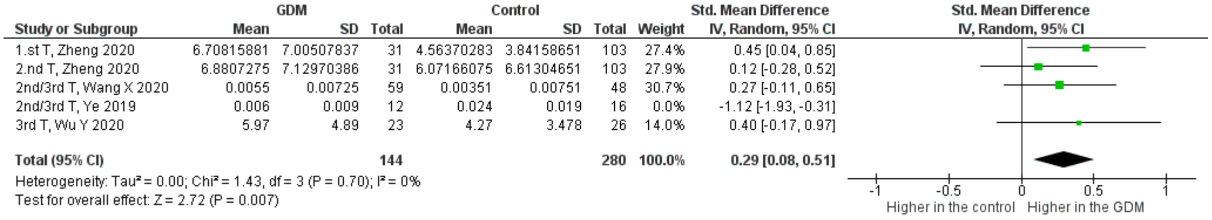

Ruminococcus

Study or Subgroup	GDM Mean	GDM SD	GDM Total	Control Mean	Control SD	Control Total	Weight	Std. Mean Difference IV, Random, 95% CI
1.st T, Zheng 2020	3.0931148	3.71401958	31	2.22015292	1.88106583	103	27.4%	0.36 [-0.05, 0.76]
2.nd T, Zheng 2020	2.88593273	2.5109828	31	2.90792611	3.58330377	103	27.7%	-0.01 [-0.41, 0.40]
2nd/3rd T, Wang X 2020	0.000724	0.00236	59	0.00106	0.00583	48	30.7%	-0.08 [-0.46, 0.30]
3rd T, Wu Y 2020	2.327	0.972	23	2.276	1.176	26	14.2%	0.05 [-0.51, 0.61]
**Total (95% CI)**			**144**			**280**	**100.0%**	**0.08 [-0.13, 0.29]**

Heterogeneity: Tau² = 0.00; Chi² = 2.68, df = 3 (P = 0.44); I² = 0%
Test for overall effect: Z = 0.73 (P = 0.46)

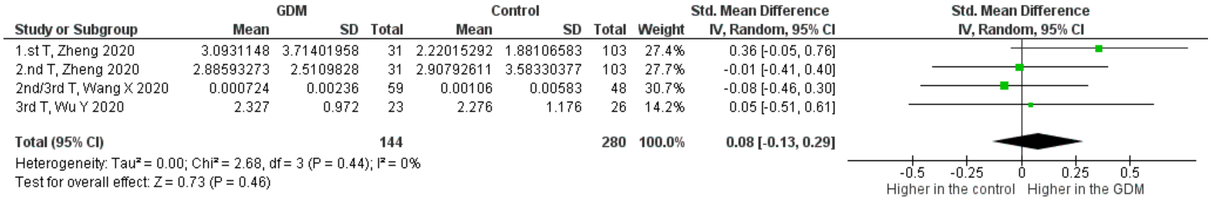

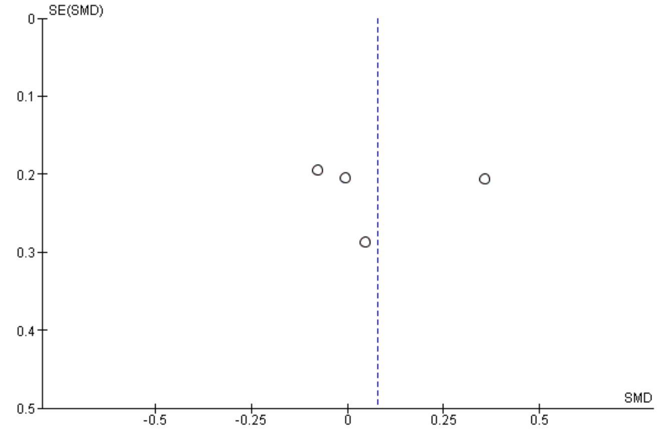

Blautia

All

Study or Subgroup	GDM Mean	GDM SD	GDM Total	Control Mean	Control SD	Control Total	Weight	Std. Mean Difference IV, Random, 95% CI
1.st T, Zheng 2020	2.80161951	1.52692453	31	2.82685097	2.00428588	103	15.8%	-0.01 [-0.41, 0.39]
2.nd T, Zheng 2020	4.69145771	3.49653452	31	4.1073442	3.5511533	103	15.8%	0.16 [-0.24, 0.57]
2nd/3rd T, Tanaka 2022	6.926	2.3575	20	6.994	3.7625	16	14.7%	-0.02 [-0.68, 0.64]
2nd/3rd T, Wang X 2020	0.255	0.213	59	0.049	0.045	48	15.7%	1.27 [0.85, 1.69]
2nd/3rd T, Xu 2020	8.048	6.841	43	5.926	5.385	81	15.9%	0.36 [-0.02, 0.73]
3rd T, Benítez-Guerrero 2022	17.27	2.764927	11	4.29	0.279708	30	7.0%	8.96 [6.81, 11.11]
3rd T, Wu Y 2020	3.758	1.891	23	4.915	3.746	26	15.1%	-0.38 [-0.94, 0.19]
**Total (95% CI)**			**218**			**407**	**100.0%**	**0.85 [0.10, 1.59]**

Heterogeneity: Tau² = 0.88; Chi² = 91.40, df = 6 (P < 0.00001); I² = 93%
Test for overall effect: Z = 2.21 (P = 0.03)

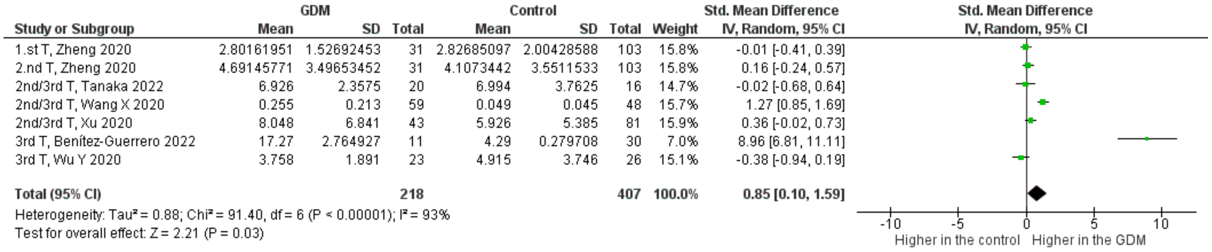

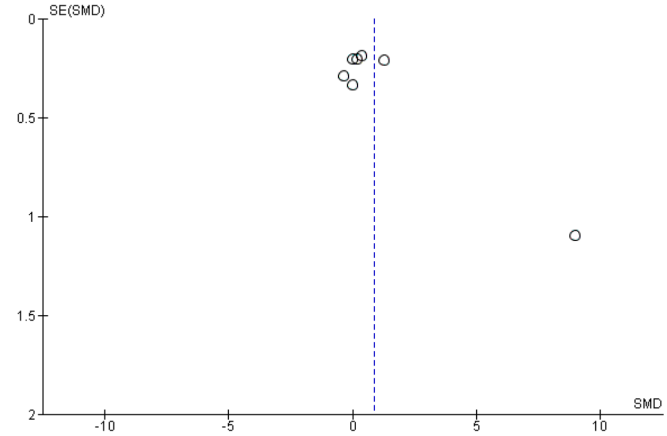

Without outlier

Study or Subgroup	GDM Mean	GDM SD	GDM Total	Control Mean	Control SD	Control Total	Weight	Std. Mean Difference IV, Random, 95% CI
1.st T, Zheng 2020	2.80161951	1.52692453	31	2.82685097	2.00428588	103	17.6%	-0.01 [-0.41, 0.39]
2.nd T, Zheng 2020	4.69145771	3.49653452	31	4.1073442	3.5511533	103	17.5%	0.16 [-0.24, 0.57]
2nd/3rd T, Tanaka 2022	6.926	2.3575	20	6.994	3.7625	16	14.2%	-0.02 [-0.68, 0.64]
2nd/3rd T, Wang X 2020	0.255	0.213	59	0.049	0.045	48	17.3%	1.27 [0.85, 1.69]
2nd/3rd T, Xu 2020	8.048	6.841	43	5.926	5.385	81	17.9%	0.36 [-0.02, 0.73]
3rd T, Benítez-Guerrero 2022	17.27	2.764927	11	4.29	0.279708	30	0.0%	8.96 [6.81, 11.11]
3rd T, Wu Y 2020	3.758	1.891	23	4.915	3.746	26	15.4%	-0.38 [-0.94, 0.19]
**Total (95% CI)**			**207**			**377**	**100.0%**	**0.25 [-0.20, 0.70]**

Heterogeneity: Tau² = 0.26; Chi² = 29.85, df = 5 (P < 0.0001); I² = 83%
Test for overall effect: Z = 1.08 (P = 0.28)

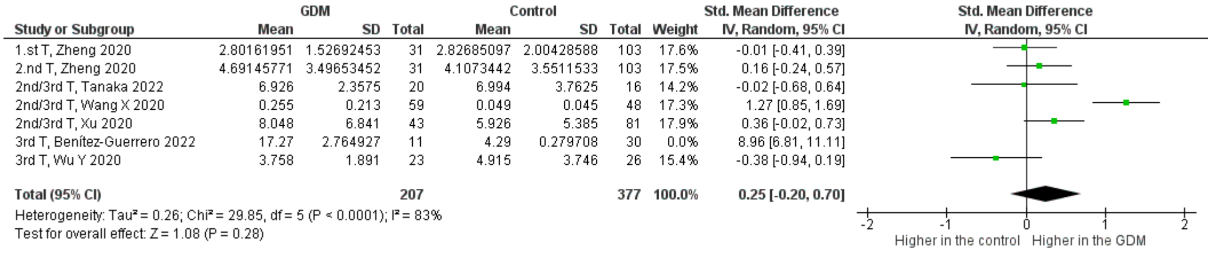

Alistipes

Study or Subgroup	GDM Mean	GDM SD	GDM Total	Control Mean	Control SD	Control Total	Weight	Std. Mean Difference IV, Random, 95% CI
1.st T, Zheng 2020	1.85655389	2.72514786	31	2.37149544	2.14572762	103	40.0%	-0.22 [-0.63, 0.18]
2.nd T, Zheng 2020	2.22360133	2.37247685	31	3.11522668	3.27004913	103	39.8%	-0.29 [-0.69, 0.12]
3rd T, Wu Y 2020	4.866	3.423	23	6.141	3.648	26	20.2%	-0.35 [-0.92, 0.21]
**Total (95% CI)**			**85**			**232**	**100.0%**	**-0.28 [-0.53, -0.02]**

Heterogeneity: Tau² = 0.00; Chi² = 0.14, df = 2 (P = 0.93); I² = 0%
Test for overall effect: Z = 2.12 (P = 0.03)

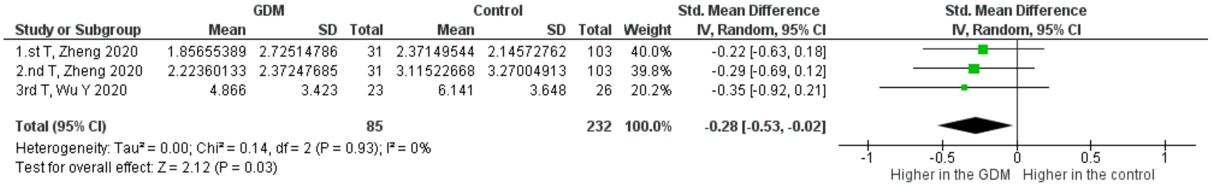

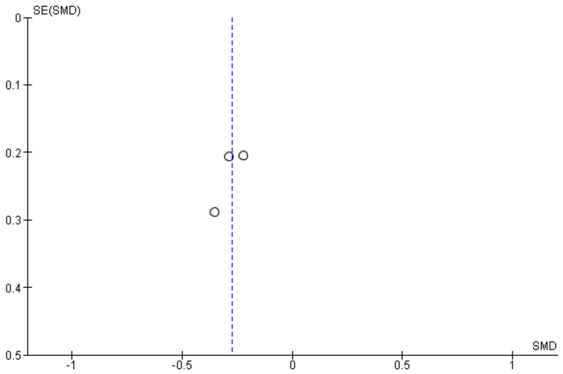

Parabacteroides

Study or Subgroup	GDM Mean	GDM SD	GDM Total	Control Mean	Control SD	Control Total	Weight	Std. Mean Difference IV, Random, 95% CI
1.st T, Zheng 2020	1.80862685	1.59066703	31	2.17714002	2.02663708	103	38.1%	-0.19 [-0.59, 0.21]
2.nd T, Zheng 2020	1.7952884	1.34074675	31	1.51480901	1.43938159	103	38.1%	0.20 [-0.21, 0.60]
3rd T, Wu Y 2020	4.219	1.915	23	5.173	3.07	26	23.9%	-0.36 [-0.93, 0.20]
**Total (95% CI)**			**85**			**232**	**100.0%**	**-0.08 [-0.40, 0.24]**

Heterogeneity: Tau² = 0.03; Chi² = 3.05, df = 2 (P = 0.22); I² = 34%
Test for overall effect: Z = 0.51 (P = 0.61)

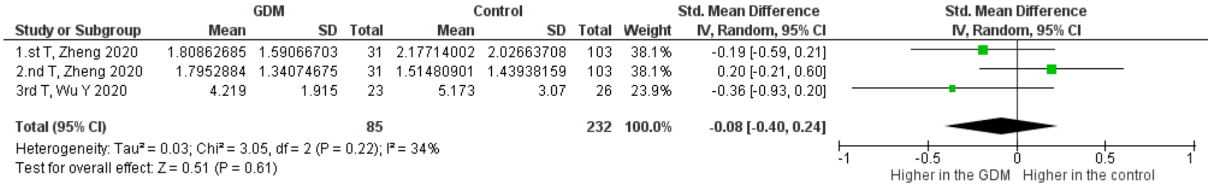

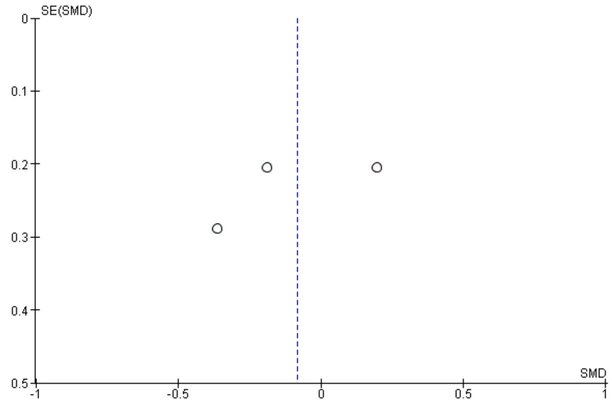

Coprococcus

Study or Subgroup	GDM Mean	GDM SD	GDM Total	Control Mean	Control SD	Control Total	Weight	Std. Mean Difference IV, Random, 95% CI
1.st T, Zheng 2020	0.79455607	0.57967808	31	1.27642236	1.09740381	103	26.4%	-0.48 [-0.88, -0.07]
2.nd T, Zheng 2020	1.16725857	1.00619117	31	1.40688903	1.20671211	103	26.6%	-0.20 [-0.61, 0.20]
2nd/3rd T, Xu 2020	5.046	4.49	43	4.311	3.31	81	28.7%	0.19 [-0.18, 0.56]
3rd T, Wu Y 2020	2.372	1.089	23	2.7	1.808	26	18.3%	-0.21 [-0.78, 0.35]
**Total (95% CI)**			**128**			**313**	**100.0%**	**-0.16 [-0.46, 0.14]**

Heterogeneity: Tau² = 0.05; Chi² = 5.94, df = 3 (P = 0.11); I² = 49%
Test for overall effect: Z = 1.07 (P = 0.29)

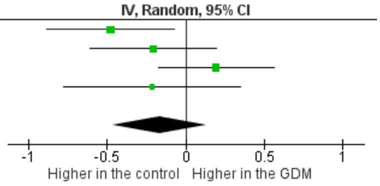

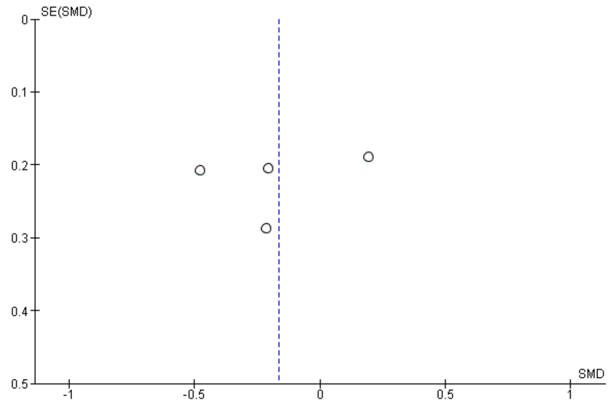

Clostridium

Study or Subgroup	GDM Mean	GDM SD	GDM Total	Control Mean	Control SD	Control Total	Weight	Std. Mean Difference IV, Random, 95% CI
1.st T, Zheng 2020	4.29096702	2.83626906	31	4.5541	3.9062	103	27.0%	-0.07 [-0.47, 0.33]
2.nd T, Zheng 2020	4.707	4.949	31	3.71899	3.3816	103	26.9%	0.26 [-0.14, 0.66]
2nd/3rd T, Xu 2020	3.758	2.026	43	4.202	2.836	81	30.2%	-0.17 [-0.54, 0.20]
3rd T, Wu Y 2020	6.827	7.267	23	4.639	3.171	26	15.9%	0.39 [-0.17, 0.96]
**Total (95% CI)**			**128**			**313**	**100.0%**	**0.06 [-0.19, 0.31]**

Heterogeneity: Tau² = 0.02; Chi² = 4.15, df = 3 (P = 0.25); I² = 28%
Test for overall effect: Z = 0.48 (P = 0.63)

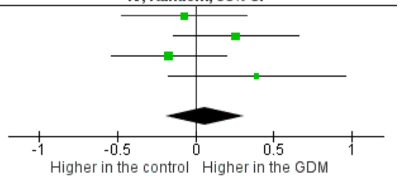

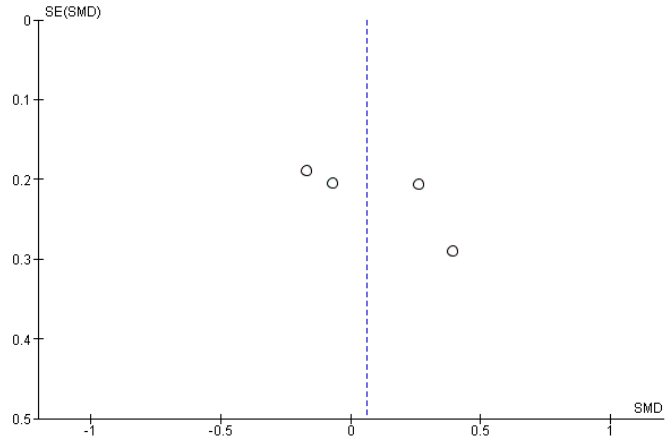

Meconium

Phylum

Acidobacteria

Study or Subgroup	GDM Mean	GDM SD	GDM Total	Control Mean	Control SD	Control Total	Weight	Std. Mean Difference IV, Random, 95% CI	Year
Hu 2013	0.09	0.2	5	0.14	0.33	9	12.4%	-0.16 [-1.26, 0.94]	2013
Su 2018	1.51	2.46	15	1.35	1.77	14	28.1%	0.07 [-0.66, 0.80]	2018
Guzzardi 2022	0.01	0.03	21	0.04	0.12	58	59.4%	-0.28 [-0.79, 0.22]	2022
**Total (95% CI)**			**41**			**81**	**100.0%**	**-0.17 [-0.56, 0.22]**	

Heterogeneity: Tau² = 0.00; Chi² = 0.63, df = 2 (P = 0.73); I² = 0%
Test for overall effect: Z = 0.86 (P = 0.39)

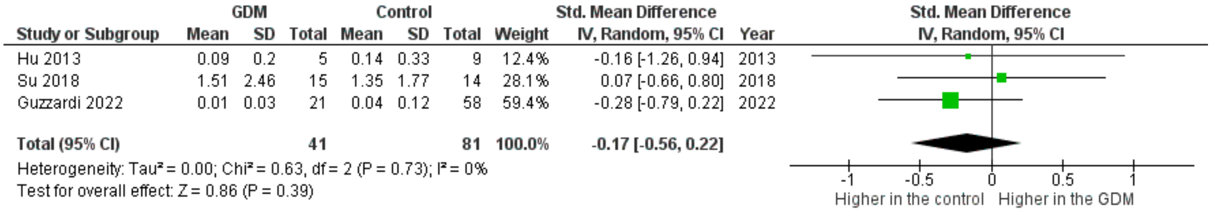

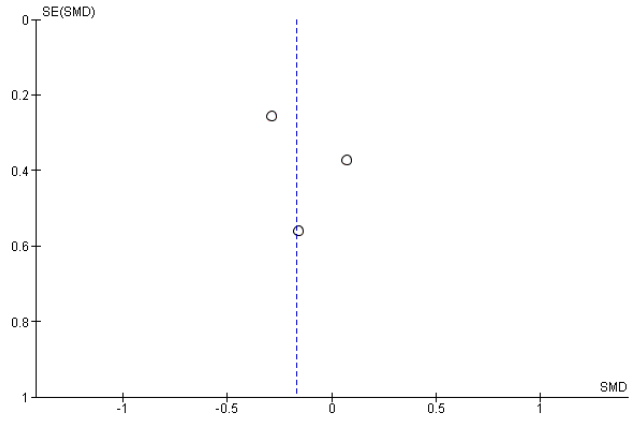

Actinobacteria

Study or Subgroup	GDM Mean	GDM SD	GDM Total	Control Mean	Control SD	Control Total	Weight	Std. Mean Difference IV, Random, 95% CI	Year
Hu 2013	2.49	4.63	5	0.9	0.92	9	18.0%	0.54 [-0.58, 1.65]	2013
Su 2018	10.79	3.49	15	7.57	3.83	14	31.4%	0.86 [0.09, 1.62]	2018
Guzzardi 2022	19.41	18.65	21	18.49	14.05	58	50.5%	0.06 [-0.44, 0.56]	2022
**Total (95% CI)**			**41**			**81**	**100.0%**	**0.40 [-0.13, 0.92]**	

Heterogeneity: Tau² = 0.08; Chi² = 3.07, df = 2 (P = 0.22); I² = 35%
Test for overall effect: Z = 1.46 (P = 0.14)

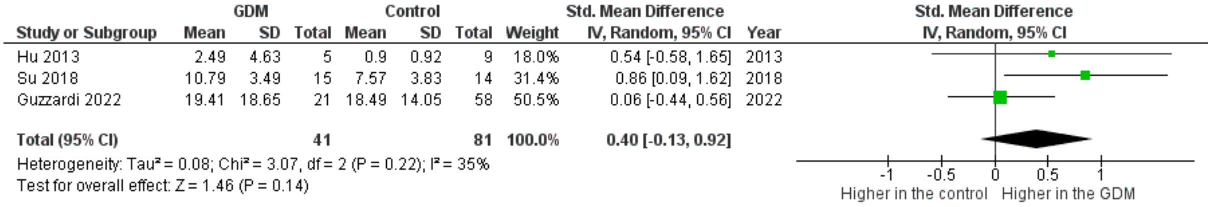

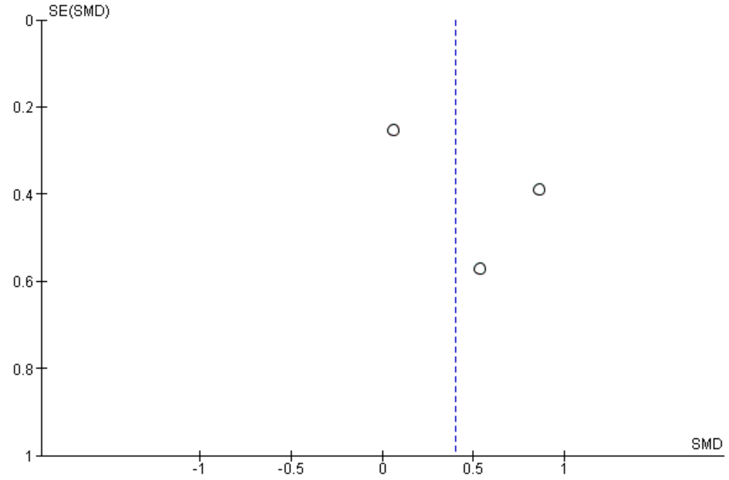

Bacteroidetes

Study or Subgroup	GDM Mean	GDM SD	GDM Total	Control Mean	Control SD	Control Total	Weight	Std. Mean Difference IV, Random, 95% CI	Year
Hu 2013	13.67	13.68	5	2.87	4.01	9	24.9%	1.18 [-0.03, 2.39]	2013
Su 2018	5.59	5.39	15	11.44	8.62	14	34.6%	-0.80 [-1.56, -0.04]	2018
Guzzardi 2022	3.77	6.18	21	4.37	1,151	58	40.5%	-0.00 [-0.50, 0.50]	2022
**Total (95% CI)**			**41**			**81**	**100.0%**	**0.02 [-0.87, 0.90]**	

Heterogeneity: Tau² = 0.44; Chi² = 7.70, df = 2 (P = 0.02); I² = 74%
Test for overall effect: Z = 0.04 (P = 0.97)

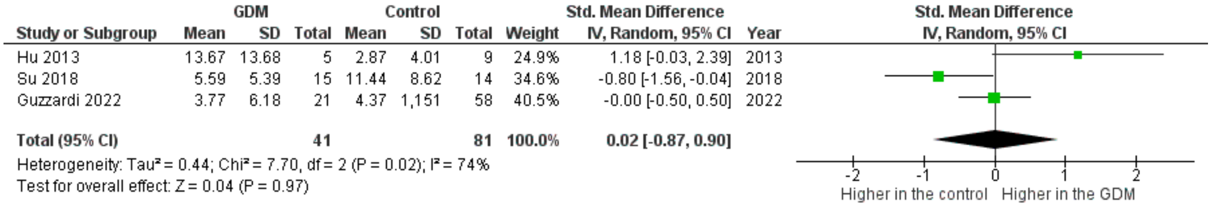

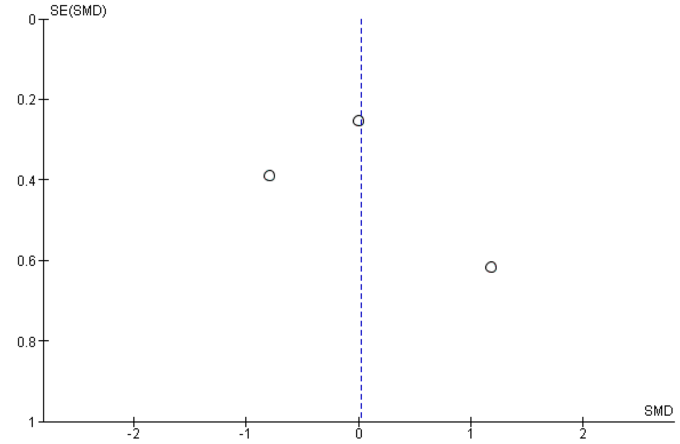

Cyanobacteria

Study or Subgroup	GDM Mean	GDM SD	GDM Total	Control Mean	Control SD	Control Total	Weight	Std. Mean Difference IV, Random, 95% CI	Year
Hu 2013	0.7	1.56	5	0.15	0.15	9	22.8%	0.57 [-0.55, 1.69]	2013
Su 2018	0.41	0.42	15	1.63	1.8	14	33.3%	-0.92 [-1.69, -0.15]	2018
Guzzardi 2022	0.46	1.15	21	1.11	3.41	58	43.9%	-0.22 [-0.72, 0.29]	2022
**Total (95% CI)**			**41**			**81**	**100.0%**	**-0.27 [-0.96, 0.42]**	

Heterogeneity: Tau² = 0.22; Chi² = 4.91, df = 2 (P = 0.09); I² = 59%
Test for overall effect: Z = 0.77 (P = 0.44)

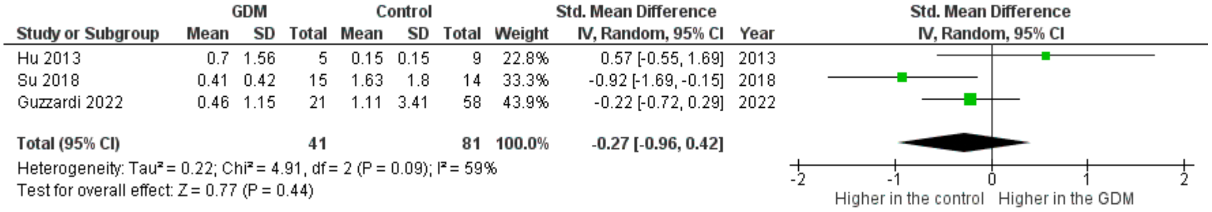

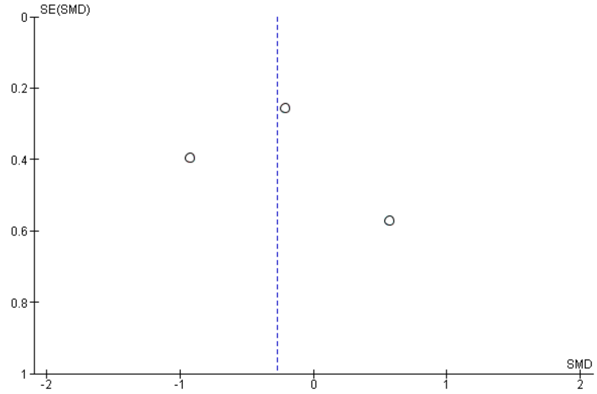

Firmicutes

Study or Subgroup	GDM Mean	GDM SD	GDM Total	Control Mean	Control SD	Control Total	Weight	Std. Mean Difference IV, Random, 95% CI	Year
Hu 2013	43.19	32.69	5	19.03	23.14	9	18.7%	0.85 [-0.31, 2.00]	2013
Su 2018	6.87	6.66	15	11.64	13.49	14	33.5%	-0.44 [-1.18, 0.30]	2018
Guzzardi 2022	49.8	25.86	21	46.73	25.2	58	47.7%	0.12 [-0.38, 0.62]	2022
**Total (95% CI)**			**41**			**81**	**100.0%**	**0.07 [-0.51, 0.65]**	

Heterogeneity: Tau² = 0.12; Chi² = 3.60, df = 2 (P = 0.17); I² = 44%
Test for overall effect: Z = 0.23 (P = 0.82)

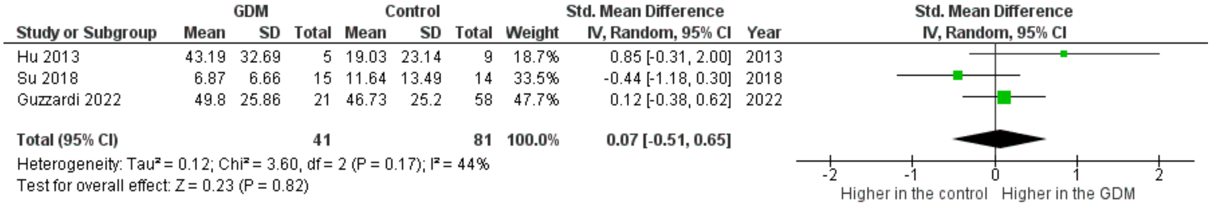

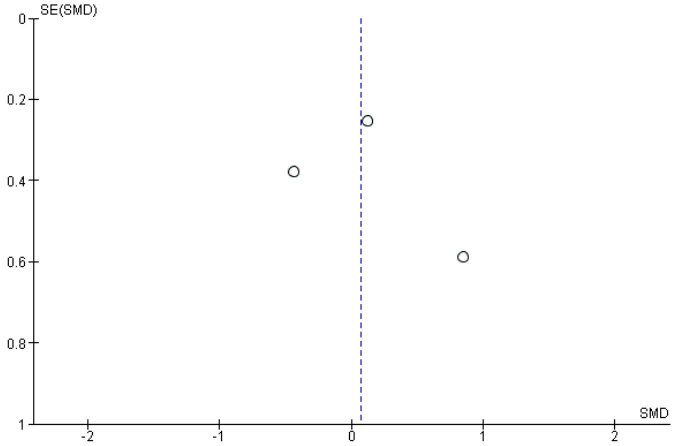

Proteobacteria

Study or Subgroup	GDM Mean	GDM SD	GDM Total	Control Mean	Control SD	Control Total	Weight	Std. Mean Difference IV, Random, 95% CI	Year
Hu 2013	34.15	36.85	5	69.13	27.6	9	22.7%	-1.06 [-2.24, 0.13]	2013
Su 2018	55.98	15.17	15	46.34	15.42	14	34.6%	0.61 [-0.14, 1.36]	2018
Guzzardi 2022	26.03	29.13	21	28	26.22	58	42.7%	-0.07 [-0.57, 0.43]	2022
**Total (95% CI)**			**41**			**81**	**100.0%**	**-0.06 [-0.81, 0.69]**	

Heterogeneity: Tau² = 0.28; Chi² = 5.70, df = 2 (P = 0.06); I² = 65%
Test for overall effect: Z = 0.15 (P = 0.88)

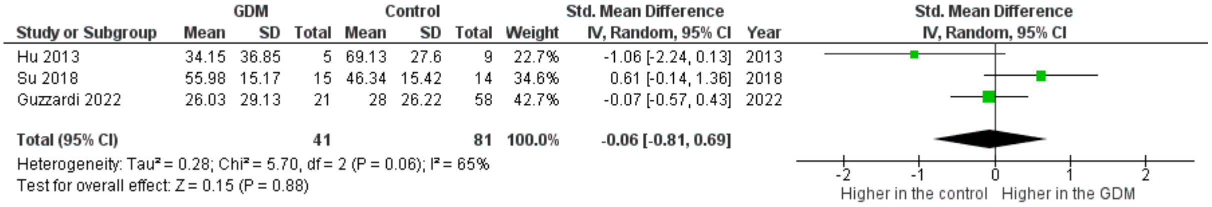

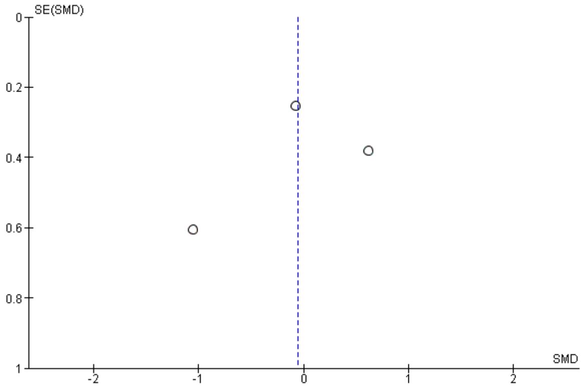

Synergistetes

Study or Subgroup	GDM Mean	GDM SD	GDM Total	Control Mean	Control SD	Control Total	Weight	Std. Mean Difference IV, Random, 95% CI	Year
Hu 2013	0.13	0.2	5	0	0	9	20.4%	1.05 [-0.13, 2.24]	2013
Su 2018	5.78	7.05	15	6.72	7.72	14	34.8%	-0.12 [-0.85, 0.61]	2018
Guzzardi 2022	0.0002	0.0007	21	0.002	0.006	58	44.8%	-0.34 [-0.85, 0.16]	2022
**Total (95% CI)**			**41**			**81**	**100.0%**	**0.02 [-0.64, 0.68]**	

Heterogeneity: Tau² = 0.19; Chi² = 4.53, df = 2 (P = 0.10); I² = 56%
Test for overall effect: Z = 0.05 (P = 0.96)

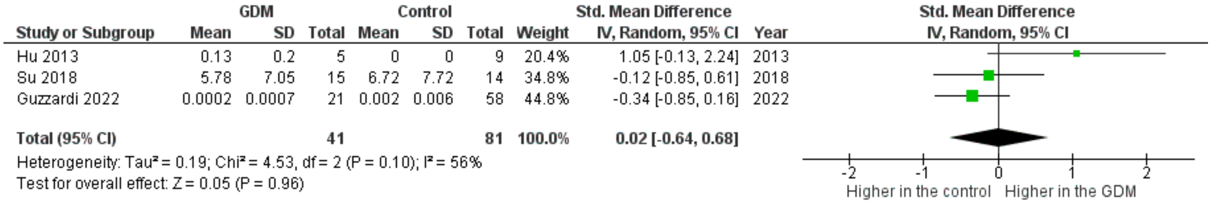

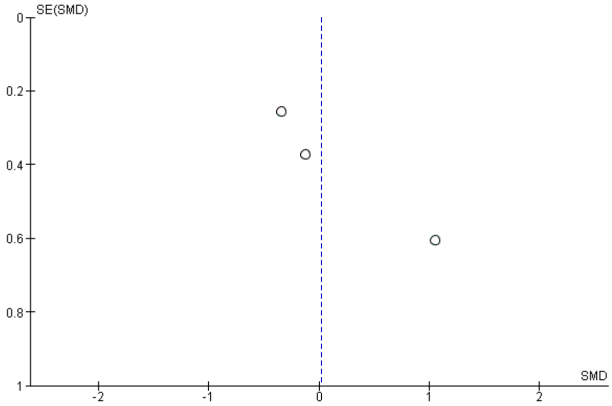

Genus

Acinetobacter

Study or Subgroup	GDM Mean	GDM SD	GDM Total	Control Mean	Control SD	Control Total	Weight	Std. Mean Difference IV, Random, 95% CI	Year
Su 2018	13.10891	9.02712	15	7.76521	5.903	14	17.8%	0.68 [-0.08, 1.43]	2018
Guzzardi 2022	0.12	0.00182748	21	0.08239592	0.10597968	58	32.7%	0.41 [-0.10, 0.91]	2022
Zhu 2022	0.08756871	0.1369472	60	0.08239592	0.10597968	60	49.5%	0.04 [-0.32, 0.40]	2022
**Total (95% CI)**			**96**			**132**	**100.0%**	**0.27 [-0.07, 0.62]**	

Heterogeneity: Tau² = 0.03; Chi² = 2.89, df = 2 (P = 0.24); I² = 31%
Test for overall effect: Z = 1.55 (P = 0.12)

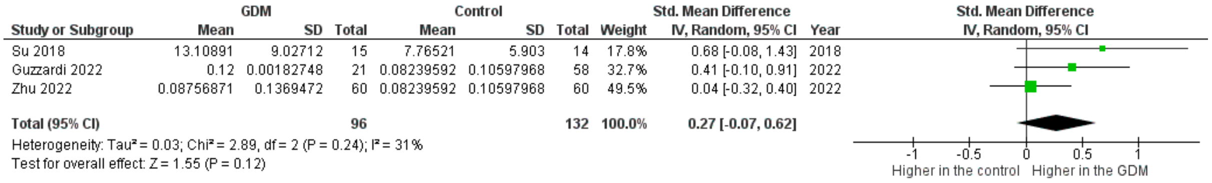

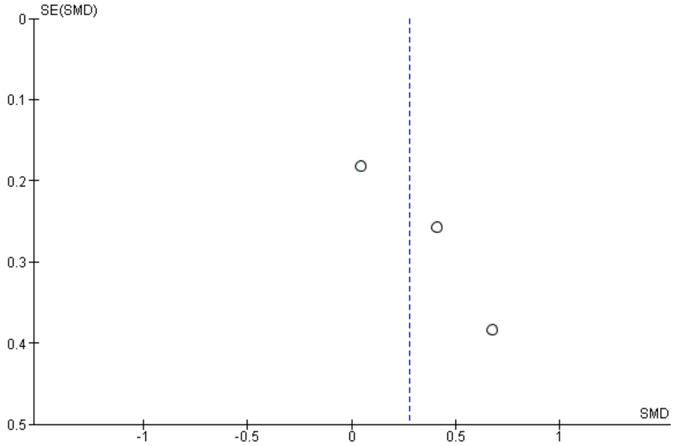

Staphylococcus

Study or Subgroup	GDM Mean	GDM SD	GDM Total	Control Mean	Control SD	Control Total	Weight	Std. Mean Difference IV, Random, 95% CI	Year
Su 2018	4.32343	5.24871	15	11.32013	21.951	14	42.6%	-0.43 [-1.17, 0.30]	2018
Guzzardi 2022	1.3	0.03163008	21	0.24	0.00535233	58	14.4%	62.61 [52.58, 72.64]	2022
Zhu 2022	0.04491656	0.1721297	60	0.00878334	0.01843025	60	43.0%	0.29 [-0.07, 0.65]	2022
**Total (95% CI)**			**96**			**132**	**100.0%**	**8.93 [4.28, 13.59]**	

Heterogeneity: Tau² = 13.08; Chi² = 151.87, df = 2 (P < 0.00001); I² = 99%
Test for overall effect: Z = 3.76 (P = 0.0002)

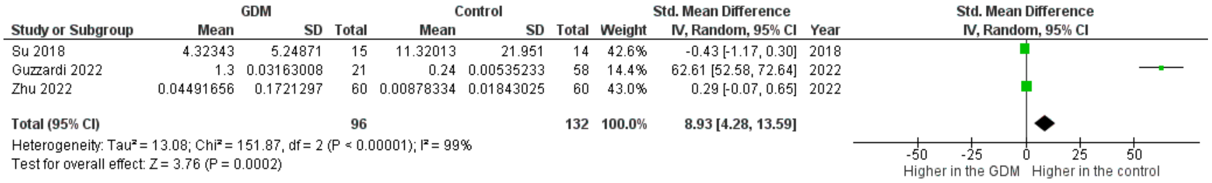

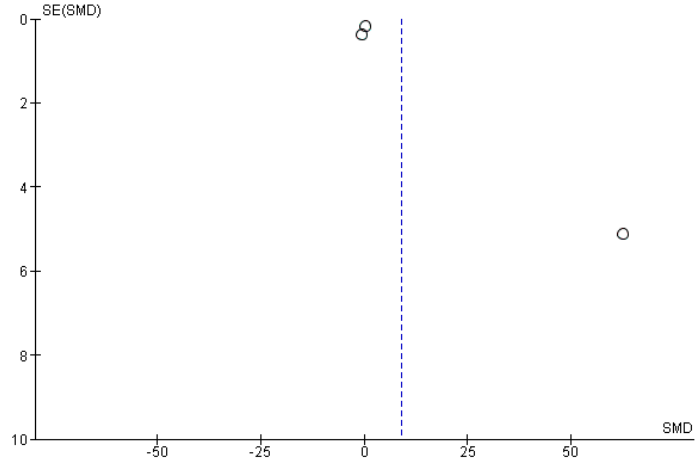

Pseudomonas

Study or Subgroup	GDM Mean	GDM SD	GDM Total	Control Mean	Control SD	Control Total	Weight	Std. Mean Difference IV, Random, 95% CI	Year
Su 2018	4.63366	2.78317	15	3.62093	2.9345	14	34.9%	0.34 [-0.39, 1.08]	2018
Guzzardi 2022	0.19	0.00326046	21	0.26	0.00426875	58	30.0%	-17.19 [-19.99, -14.40]	2022
Zhu 2022	0.06746793	0.11670058	60	0.10869247	0.15219395	60	35.2%	-0.30 [-0.66, 0.06]	2022
**Total (95% CI)**			**96**			**132**	**100.0%**	**-5.14 [-9.08, -1.20]**	

Heterogeneity: Tau² = 11.45; Chi² = 142.88, df = 2 (P < 0.00001); I² = 99%
Test for overall effect: Z = 2.56 (P = 0.01)

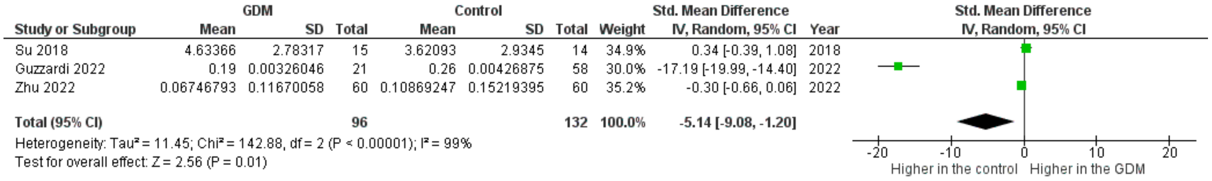

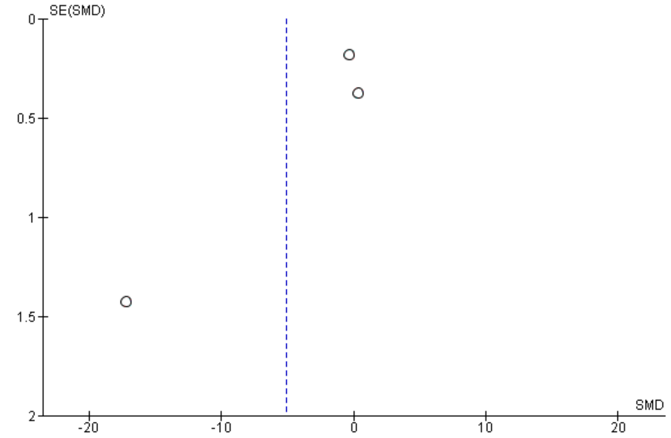

Enhydrobacter

Study or Subgroup	GDM Mean	GDM SD	GDM Total	Control Mean	Control SD	Control Total	Weight	Std. Mean Difference IV, Random, 95% CI	Year
Su 2018	2.15182	1.37979	15	3.0099	2.396	14	38.9%	-0.43 [-1.17, 0.31]	2018
Zhu 2022	0.01	0.0002229	21	0.03	0.00058387	58	21.9%	-38.45 [-44.62, -32.28]	2022
Guzzardi 2022	0.00147	0.00248526	60	0.00531357	0.01902893	60	39.2%	-0.28 [-0.64, 0.08]	2022
**Total (95% CI)**			**96**			**132**	**100.0%**	**-8.71 [-13.05, -4.36]**	

Heterogeneity: Tau² = 12.49; Chi² = 146.47, df = 2 (P < 0.00001); I² = 99%
Test for overall effect: Z = 3.93 (P < 0.0001)

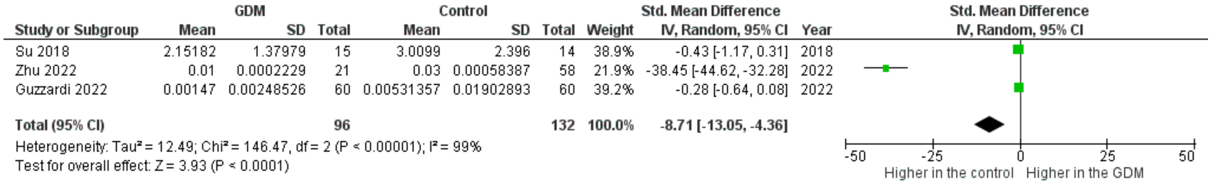

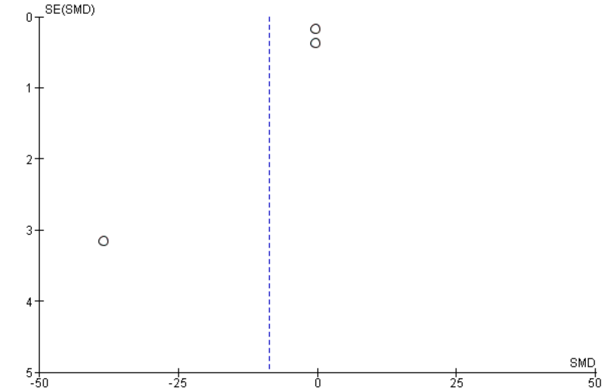

Serratia

Study or Subgroup	GDM Mean	GDM SD	GDM Total	Control Mean	Control SD	Control Total	Weight	Std. Mean Difference IV, Random, 95% CI	Year
Su 2018	3.88119	2.64276	15	1.84158	0.8907	14	21.4%	0.99 [0.21, 1.77]	2018
Guzzardi 2022	0	0.00000455	21	0	0.00011287	58	34.7%	0.00 [-0.50, 0.50]	2022
Zhu 2022	0.00890469	0.02651192	60	0.00387326	0.00389243	60	43.9%	0.26 [-0.10, 0.62]	2022
**Total (95% CI)**			**96**			**132**	**100.0%**	**0.33 [-0.12, 0.77]**	

Heterogeneity: Tau² = 0.08; Chi² = 4.42, df = 2 (P = 0.11); I² = 55%
Test for overall effect: Z = 1.44 (P = 0.15)

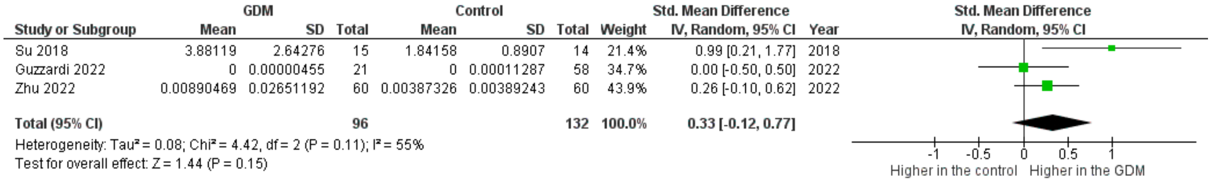

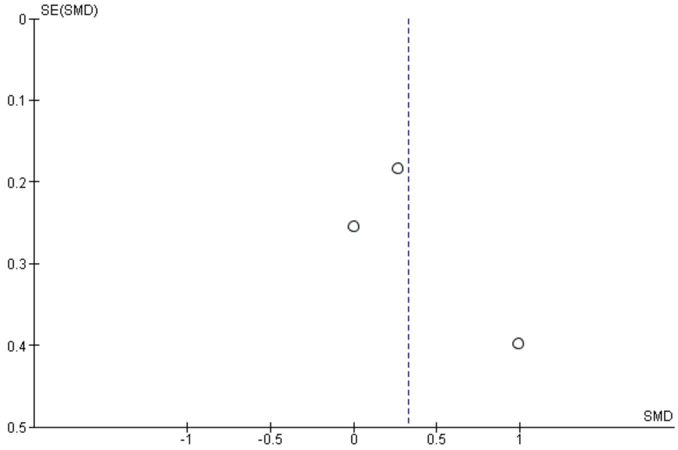

Paracoccus

Study or Subgroup	GDM Mean	GDM SD	GDM Total	Control Mean	Control SD	Control Total	Weight	Std. Mean Difference IV, Random, 95% CI	Year
Hu 2013	1.46535	0.89125	15	1.40814	0.7761	14	40.6%	0.07 [-0.66, 0.79]	2013
Su 2018	0.01	0.00020247	21	0.07	0.00144604	58	18.5%	-47.59 [-55.22, -39.96]	2018
Zhu 2022	0.00151654	0.00309332	60	0.00219368	0.004547	60	40.9%	-0.17 [-0.53, 0.19]	2022
**Total (95% CI)**			**96**			**132**	**100.0%**	**-8.86 [-13.29, -4.43]**	

Heterogeneity: Tau² = 12.48; Chi² = 148.75, df = 2 (P < 0.00001); I² = 99%
Test for overall effect: Z = 3.92 (P < 0.0001)

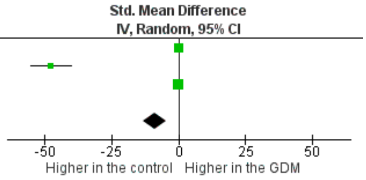

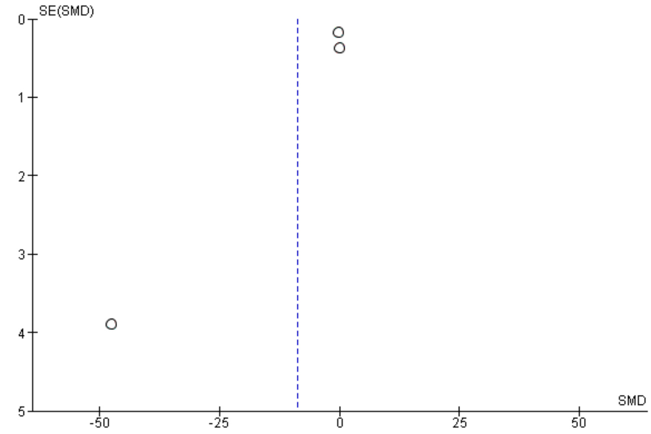

Supplementary Material File S3 - Article Bias

Cohort Studies

Study	Zhang, Y., et al., 2021 [43]	Guzzardi, et al., 2022 [46]	Wang, J., et al., 2018 [35]	Wei, et al., 2022 [37]	Su, M., et al., 2018 [31]	Zhu, et al., 2022 [45]
1. Was the research question or objective in this paper clearly stated?	yes	yes	yes	yes	yes	yes
2. Was the study population clearly specified and defined?	yes	yes	yes	yes	yes	yes
3. Was the participation rate of eligible persons at least 50%?	yes	NR	NR	yes	NR	NR
4. Were all the subjects selected or recruited from the same or similar populations (including the same time period)? Were inclusion and exclusion criteria for being in the study prespecified and applied uniformly to all participants?	yes	yes	yes	yes	yes	yes
5. Was a sample size justification, power description, or variance and effect estimates provided?	no	no	no	no	no	no
6. For the analyses in this paper, were the exposure(s) of interest measured prior to the outcome(s) being measured?	yes	yes	yes	yes	yes	yes
7. Was the timeframe sufficient so that one could reasonably expect to see an association between exposure and outcome if it existed?	yes	yes	yes	yes	yes	yes
8. For exposures that can vary in amount or level, did the study examine different levels of the exposure as related to the outcome (e.g., categories of exposure, or exposure measured as continuous variable)?	NA	NA	NA	NA	yes	NA
9. Were the exposure measures (independent variables) clearly defined, valid, reliable, and implemented consistently across all study participants?	yes	yes	yes	yes	yes	yes
10. Was the exposure(s) assessed more than once over time?	no	yes	no	no	no	no
11. Were the outcome measures (dependent variables) clearly defined, valid, reliable, and implemented consistently across all study participants?	yes	yes	yes	yes	yes	yes
12. Were the outcome assessors blinded to the exposure status of participants?	NR	NR	NR	NR	NR	NR

13. Was loss to follow-up after baseline 20% or less?	NA	yes	NA	NA	no	yes
14. Were key potential confounding variables measured and adjusted statistically for their impact on the relationship between exposure(s) and outcome(s)?	Yes	yes	yes	yes	yes	yes
Rater:	good	good	good	good	good	good

National Institutes of Health (https://www.nhlbi.nih.gov/health-topics/study-quality-assessment-tools).

[26]

Case Control Studies

**Study**	**Zheng, W., et al., 2020 [44]**	**Hu, P., et al., 2021 [30]**	**Xu, Y., et al., 2020 [40]**	**Wang, X., et al., 2020 [36]**	**Hu, J., et al., 2013 [29]**	**Benítez-Guerrero, et al., 2022 [28]**	**Wu, Y., et al., 2020 [39]**	**Su, Y., et al., 2021 [32]**	**Ye, et al., 2019 [41]**	**Bassols, et al., 2016 [27]**	**Tang, N., et al., 2020 [34]**
1. Was the research question or objective in this paper clearly stated and appropriate?	yes	yes	yes	yes	yes	yes	yes	yes	yes	yes	yes
2. Was the study population clearly specified and defined?	yes	yes	yes	no	yes	yes	yes	yes	yes	yes	yes
3. Did the authors include a sample size justification?	no	no	no	no	no	no	no	no	no	no	no

4. Were controls selected or recruited from the same or similar population that gave rise to the cases (including the same timeframe)?	yes	yes	yes	yes	yes	yes	yes	yes	yes	yes	yes
5. Were the definitions, inclusion and exclusion criteria, algorithms or processes used to identify or select cases and controls valid, reliable, and implemented consistently across all study participants?	yes	yes	yes	yes	yes	yes	yes	yes	yes	yes	yes
6. Were the cases clearly defined and differentiated from controls?	yes	yes	yes	yes	yes	yes	yes	yes	yes	yes	yes
7. If less than 100 percent of eligible cases and/or controls were selected for the study, were the cases and/or controls randomly selected from those eligible?	NA	NR	NR	NR	NA	NR	NR	NA	NA	NA	NR
8. Was there use of concurrent controls?	yes	yes	yes	yes	yes	yes	yes	yes	yes	yes	yes
9. Were the investigators able to confirm that the exposure/risk occurred prior to the development of the condition or event that defined a participant as a case?	yes	yes	yes	yes	yes	yes	yes	no	no	yes	yes

10. Were the measures of exposure/risk clearly defined, valid, reliable, and implemented consistently (including the same time period) across all study participants?	yes	yes	yes	yes	yes	yes	yes	yes	yes	yes	NR
11. Were the assessors of exposure/risk blinded to the case or control status of participants?	NR	NR	NR	NR	NR	NR	NR	NR	NR	NR	NR
12. Were key potential confounding variables measured and adjusted statistically in the analyses? If matching was used, did the investigators account for matching during study analysis?	yes	yes	yes	yes	yes	yes	yes	yes	yes	yes	yes
rate	good	good	good	good	good	good	good	good	good	good	fair

National Institutes of Health (https://www.nhlbi.nih.gov/health-topics/study-quality-assessment-tools).

[26]
